# Antibiotic-Induced Perturbations Are Manifested in the Dominant Intestinal Bacterial Phyla of Atlantic Salmon

**DOI:** 10.3390/microorganisms7080233

**Published:** 2019-08-02

**Authors:** Shruti Gupta, Jorge Fernandes, Viswanath Kiron

**Affiliations:** Faculty of Biosciences and Aquaculture, Nord University, 8049 Bodø, Norway

**Keywords:** fish, *Salmo salar*, antibiotics, florfenicol, oxolinic acid, intestinal bacteria, microbiota, high-throughput amplicon sequencing

## Abstract

The intestinal microbiota of certain farmed fish are often exposed to antimicrobial substances, such as antibiotics, that are used to prevent and treat bacterial diseases. Antibiotics that kill or inhibit the growth of harmful microbes can rapidly alter intestinal microbial diversity and composition, with potential effects on the host health. In this study, we have elucidated the impact of two antibiotics, florfenicol and oxolinic acid, by employing a high-throughput 16S rRNA gene amplicon sequencing technique on the distal and mid intestinal microbial communities of Atlantic salmon (*Salmo salar*). For this, Atlantic salmon were offered diets with or without antibiotics. We then investigated the bacterial communities in the intestinal mucus of the fish. Our results showed that antibiotic exposure shifts the intestinal microbial profile differentially. In addition, the bacterial compositions of the control and antibiotic-fed groups were significantly different. Antibiotic feeding altered the composition and abundance of the dominant bacterial phyla, namely Proteobacteria, Actinobacteria, Firmicutes, Spirochaetes, Bacteroidetes, Tenericutes, and Thermotogae. The bacterial association network analysis also indicated the differential pattern of co-occurrence of bacteria in the three study groups. The results regarding the differences in the structure and association of the intestinal microbiota of Atlantic salmon after florfenicol and oxolinic acid feeding can be employed to attenuate the adverse effects of antibiotic feeding on fish.

## 1. Introduction

Antibiotics either kill pathogenic bacteria or inhibit their growth. Although antibiotic administration is intended to help the host fight infections, it can have a detrimental effect on the commensal gut microbiota of the host. The gastrointestinal tract (GIT) is home to the gut microbiota, which is an ecological community of microorganisms that can be considered commensal, symbiotic, and pathogenic [1,2]. The microbial assemblage that colonizes the GIT includes many bacterial species, as well as other microorganisms such as fungi, viruses, and archaea [3]. The thousands of years of coevolution of the microbes and their hosts has helped to establish a complex and mutually beneficial relationship between them [4]. In healthy humans, the gut bacterial population that is the most dense and extremely diverse is found in the large intestine of the GIT [5] and offers various functions, many of which provide health benefits, including maturation of the immune system [6], immune homeostasis, and health maintenance [7]. Other functions of commensal bacteria that have significant consequences on health include biosynthesis of microbial amino acids [8], fermentation of nondigestible dietary carbohydrates into absorbable bioactive metabolites [9,10], vitamin synthesis [11], and pathogen displacement [12].

Antibiotic-induced perturbations in the established gut microbial community may result in dysbiosis that could culminate in the ill health of the host. The dose/duration and mode of action of the antibiotics and the degree of resistance exerted by the gut microbial community are the factors that govern the extent of the detrimental effects of antibiotics on the commensal organisms [13]. An imbalance in the gut microbial composition can affect the interplay between these microbes and the host, resulting in immune-mediated diseases [14]. Several studies have confirmed that antibiotic exposure rapidly alters gut microbiome composition [15], causing an imbalance in its stability. 

Although the global use of antibiotics in food-producing sectors continues to escalate, measures are being taken in the aquaculture sector to avoid the issues related to antimicrobial resistance. However, intensive farming is associated with infectious diseases. Therefore, in aquaculture, antibiotics are administered, as required, for short periods of time [16]. In countries like Norway, use of antibiotics has been dramatically reduced because of the advent of effective vaccines. Nevertheless, in many developing countries, farmers feed large amounts of antibiotics to animals, and this approach has caused problems and concerns, such as antimicrobial resistance and food safety risks. Among the antimicrobials employed, florfenicol (FFC) is by far the most commonly and frequently used antibacterial agent in Atlantic salmon farms [17]. Oxolinic acid (OA) is another antibiotic that salmon are occasionally exposed to [18]. However, its use has decreased compared to FFC [17]. FFC is an amphenicol, and members of this group are broad-spectrum (i.e., they are effective against both pathogenic and symbiotic bacteria) bacteriostatic antibiotics, and these chemicals decrease bacterial growth mainly by inhibiting protein synthesis [19]. OA is a first generation quinolone [20]. Members of the quinolones are broad-spectrum bactericidal antibiotics that are capable of killing infectious bacteria [21] by affecting their DNA metabolism through inhibiting the activities of two bacterial enzymes, DNA gyrase and topoisomerase IV, which leads to DNA fragmentation [22,23]. All quinolones can exert both bactericidal [24] and bacteriostatic effects—when bacteriostatic, they target the DNA replication process [25]. Depending on the dose, most antimicrobials can exhibit bactericidal and bacteriostatic properties. A recent study has linked higher doses of FFC and occurrence of antibiotic-resistant bacteria in the gut microbiota of farmed Atlantic salmon [26]. However, such a correlation based on OA feeding has not yet been described. 

The effects of antimicrobials have not been adequately addressed through necessary scientific research, not to mention their impact on the intestinal microbial composition of Atlantic salmon, especially when feeds are employed as the antibiotic delivery vehicles. In this study, we examined the effects of FFC and OA on the intestinal microbiota of Atlantic salmon offered feeds with or without the two antibiotics, and we found differences in the diversity and predicted associations of the mucus bacteria. 

## 2. Materials and Methods

### 2.1. Ethics Statement

We obtained approval from the Norwegian Animal Research Authority, FDU (Forsøksdyrutvalget ID-7898) before initiating the study. The fish were given enough time to acclimatize to the rearing facility. All fish used in this study received the same commercial (control) feed during the acclimation period. Furthermore, the safety procedures at the Research Station of Nord University (Norway) were followed during the experiment.

### 2.2. Experimental Fish, Rearing Conditions and Antibiotic Dosing

For the 12 days feeding trial, Atlantic salmon (25 per tank) of initial average weight 321.9 ± 36.2 g were reared in 800 L tanks in a flow-through sea water system. Three groups of fish received commercial feeds coated with (florfenicol (FFC)—F; oxolinic acid (OA)—O) or without antibiotics (Control—C) twice a day. Automatic feeders (Arvo-Teck, Huutokoski, Finland) were employed to deliver the feeds at the rate of 0.5% body weight. The dose of FFC and OA per fish was 2 g/kg and 5 g/kg, respectively, as given in Felleskatalogen [26,27]. The two antibiotics were administered as per the recommendation of the European Agency for the Evaluation of Medicinal Products [28]. The water flow rate, temperature, salinity, and O_2_ levels in the tanks were 800 L/h, 6.7–7.1 °C, 32 ppt, and >85% saturation measured at the outlet, respectively. The fish were maintained under a 24 h light regime.

### 2.3. Sampling Strategy

We sampled the mucus from the distal intestine (DI) and mid intestine (MI) of the experimental fish at the end of the feeding regime. After euthanizing the fish with 160 mg/L of MS222 tricaine methanesulfonate (Argent Chemical Laboratories, Redmond, WA, USA), the body surface of the fish was cleaned using 70% ethanol. Thereafter, the GIT was dissected out, and the DI and MI regions were carefully separated. Next, the content was removed using sterile forceps, and the intestinal mucus was collected (n = 9) using sterile glass slides into cryotubes and stored at −80 °C. Furthermore, biofilm samples were scraped from the walls of the rearing tanks (n = 3). The tank biofilm samples were also stored at −80 °C. 

The sample abbreviations used are: (i) fish samples—control distal intestine mucus (CDM), FFC distal intestine mucus (FDM), OA distal intestine mucus (ODM), control mid intestine mucus (CMM), FFC mid intestine mucus (FMM), OA mid intestine mucus (OMM), (ii) environmental samples—control tank biofilm (CB), FFC tank biofilm (FB), OA tank biofilm (OB).

### 2.4. Bacterial DNA Isolation, PCR Amplification, 16S rRNA Gene Amplicon Library Preparation and Sequencing

We first extracted the total genomic DNA from the fish and environmental samples using the Quick-DNA™ Fecal/Soil Microbe 96 kit (Zymo Research, Irvine, CA, USA). The quality of the isolated DNA was checked on 1.2% (w/v) agarose gel and quantified using a Qubit 3.0 fluorometer (Life Technologies, Carlsbad, CA, USA).

To describe the antibiotic-induced perturbations in the intestinal microbiota of the fish, we amplified the same regions (V3–V4) of the bacterial 16S rRNA gene, as reported in Gupta et al. [29]. The dual-index strategy described by Kozich et al. [30] was adopted for sequencing. The standardized PCR reaction, as described in our previous paper [29], was used to amplify the selected regions of 16S rRNA gene of the DNA. The amplicons were pooled and visualized on 1.2% (w/v) agarose gel stained with SYBER^®®^ Safe, to check the size of the amplified products. The negative PCR control did not indicate any positive amplification. The ZR-96 Zymoclean™ Gel DNA Recovery Kit (Zymo Research) was used to purify the amplicons (~550 bp). A KAPA Library Quantification Kit (KAPA Biosystems, Boston, MA, USA) was used to quantify the purified amplicon libraries. Later on, each amplicon library was normalized to an equimolar concentration (3 nM), which was validated on the TapeStation (Agilent Biosystems, Santa Clara, CA, USA). For sequencing, the normalized amplicon library pool was further diluted to 12 pM and spiked with equimolar 10% PhiX control. Next, paired-end sequencing was performed using the 600 cycle v3 sequencing kit on the Illumina MiSeq sequencer (Illumina, San Diego, CA, USA). 

### 2.5. Bioinformatic Analysis of the 16S rRNA Gene Sequence Data

#### 2.5.1. Sequence Data Quality Check

As the first step in the 16S rRNA gene sequence data analysis, the quality of the raw reads was checked using FastQC [31], and the reads with a Phred quality score (Q) ≤ 15 were discarded. Only the forward reads (R1) corresponding to the V3 region of 16S rRNA gene were employed for subsequent analyses, because they were of better quality than the reverse reads (R2) corresponding to the V4 region. 

#### 2.5.2. Sequence Data Processing

The bioinformatic pipeline UPARSE (USEARCH version 9.2.64) by Edgar [32] was used for the sequence data processing. For this, the raw FastQ files that were input into the UPARSE pipeline were truncated to 150 bp and then quality filtered. Furthermore, using the UCHIME algorithm [33], chimeric sequences were removed. The quality-filtered sequences were clustered at a 97% sequence similarity level to generate the operational taxonomic units (OTUs). All the amplicon sequences were truncated to the same length for OTU clustering. The 16S rRNA Ribosomal Database Project training set with species names v16 that uses the SINTAX algorithm was employed to assign the taxonomic ranks to the OTUs [34], using a bootstrap cutoff value of 0.5. The OTUs with a confidence score of <1 at the domain level were removed. As reported in our previous paper [29], we removed the phyla Cyanobacteria and Chlorophyta. The downstream analyses were performed on the rarified OTU count data of three sets based on the sample type, namely the DI mucus, MI mucus, and tank biofilm samples. 

Furthermore, only nine fish per group were considered for the downstream analyses, to ensure that the samples across the different tissues were from the same fish. In total, we analyzed 63 samples, including the tank biofilm samples.

#### 2.5.3. Accession Number

We have deposited the raw 16S rRNA gene sequence data in the European Nucleotide Archive (ENA); PRJEB31723.

#### 2.5.4. Sequence Data Analysis to Understand the Gut Microbial Diversity and Composition

We employed customized R codes in RStudio v3.5.0 (R Development Core Team, 2018) to assess the gut microbial diversity and composition. Functions in different R packages—‘iNEXT’ v2.0.12 [35], ‘phyloseq’ v1.22.3 [36], ‘ggplot2′ v2.2.1 [37], and ‘microbiome’ v1.0.2 [38]—were used to make the plots. The alpha diversities were calculated based on the formula suggested by Jost [39]. Beta diversity was examined by conducting double principal coordinate analysis (DPCoA) for fish and biofilm samples [40].

#### 2.5.5. Statistical Analyses of the Sequence Data

R studio v3.5.0 was used to perform the statistical analysis of the sequencing data. To detect significant differences in alpha diversity, we employed a Kruskal–Wallis test followed by Dunn’s test. After checking the assumption of heterogeneity in dispersions, we employed Adonis followed by pairwise comparisons (999 permutations) to understand the significant dissimilarities of both the intestinal and tank biofilm communities; statistical significances are reported at *p* < 0.05 and statistical trends at *p* ≤ 0.15. Furthermore, the ‘ANCOM’ v1.1-3 R package [41] detected the differentially abundant OTUs.

### 2.6. Microbial Association Graph Construction and Network Topology Inference

We used association network analysis to explore the associations between the OTUs. To generate the single-domain bacterial association network, we used ‘SPIEC-EASI’ v1.0.2 R package (SParse InversE Covariance Estimation for Ecological Association Inference), as described in Gupta, Fečkaninová, Lokesh, Koščová, Sørensen, Fernandes and Kiron [29]. The latest version of SPEIC-EASI allows analysis with fewer OTUs than we have employed before [42]; the co-occurrence microbial networks were constructed using the top 90 OTUs for DI and the top 150 OTUs for MI. The functions of the R package ‘igraph’ v1.2.1 and ggplot2 commands were utilized to customize the network plots. We analyzed the node degrees and betweenness of the control and antibiotic-fed groups using a Kruskal–Wallis test followed by Dunn’s test.

## 3. Results

### 3.1. Sequence Data and Analyses Strategy

We analyzed the 16S rRNA V3 amplicon sequences of the intestinal bacterial communities of 54 samples. Twenty-seven were of intestinal mucus samples of each DI and MI, and the remaining nine were of the tank biofilm samples. We obtained a total of 25,673,984 high-quality reads that were clustered into 1380 OTUs at a 97% identity threshold. The reads were rarified based on sample size, the saturation point being 10,044. Rarefied data was employed to assess most of the underlying microbial diversity (Figure S1A–C).

We employed diversity metrics, taxonomic composition, and relative abundances of the bacterial taxa to describe the differences in the DI and MI mucus bacterial communities of the antibiotic-fed fish compared to the control fish.

### 3.2. Changes in the Microbial Diversity of the Intestinal Mucus and Environmental Microbiota

**Figure 1:**
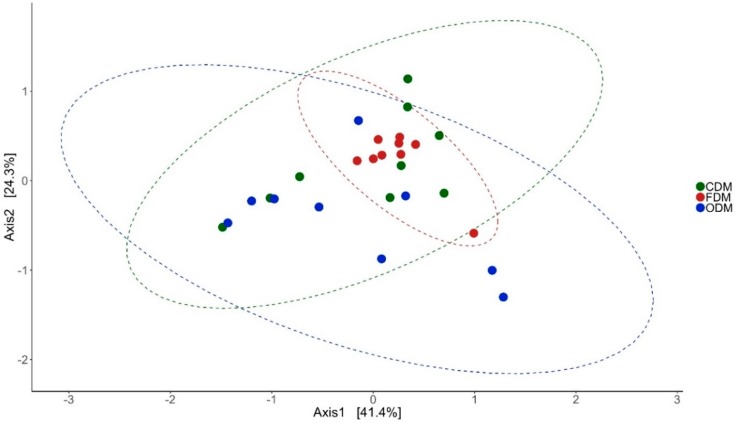
Differences in the beta diversity of the bacterial communities present in the distal intestine mucus of Atlantic salmon. The data in the ellipse-enclosed areas come from a multivariate normal distribution. The codes for the mucus samples are as follows: control group, CDM; florfenicol-fed group, FDM; oxolinic-acid-fed group, ODM.

**Figure 2:**
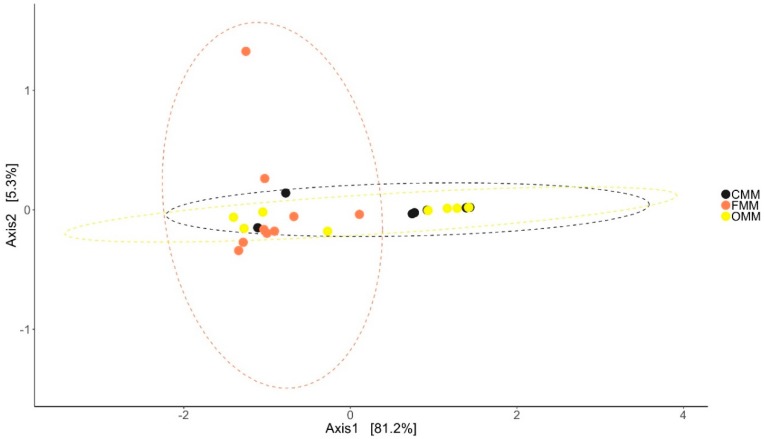
Differences in the beta diversity of the bacterial communities present in the mid intestine mucus of Atlantic salmon. The data in the ellipse-enclosed areas come from a multivariate normal distribution. The codes for the mucus samples are as follows: control group, CMM; florfenicol-fed group, FMM; oxolinic-acid-fed group, OMM.

Antibiotic feeding increased the species richness and diversity of the bacterial community in the DI and MI of the fish. The species richness was found to be higher in the DI of antibiotic-fed groups (*p* < 0.05 and *p* < 0.15). In MI, the species richness was higher only in the F-fed group. We observed significant differences in the effective number of common and dominant OTUs in the DI of the antibiotic-fed groups. Comparison of Faith’s phylogenetic diversity (PD) of both the DI and MI (Table 1) revealed differences among the groups. Please see Table 1 for the information regarding alpha diversity. Weighted unifrac distance-based PCoA revealed that the beta diversity of the bacterial communities was different; the differences between F- and O-fed groups were statistically significant (DI: Figure 1: F statistic = 2.277, R^2^ = 0.159, *p* = 0.028; MI: Figure 2: F statistic = 5.64, R^2^ = 0.32, *p* = 0.011).

The beta diversity of the bacterial communities of the biofilm samples was also analyzed. Although the bacterial communities of the three tank biofilm samples were not different (Figure S2A, F statistic = 0.76, R^2^ = 0.20, *p* = 0.538), the bacterial communities in the biofilm were significantly different from those of the fish (Figure S2B–G).

### 3.3. Changes in the Intestinal Mucus Bacterial Composition, Influenced by Antibiotics

**Figure 3:**
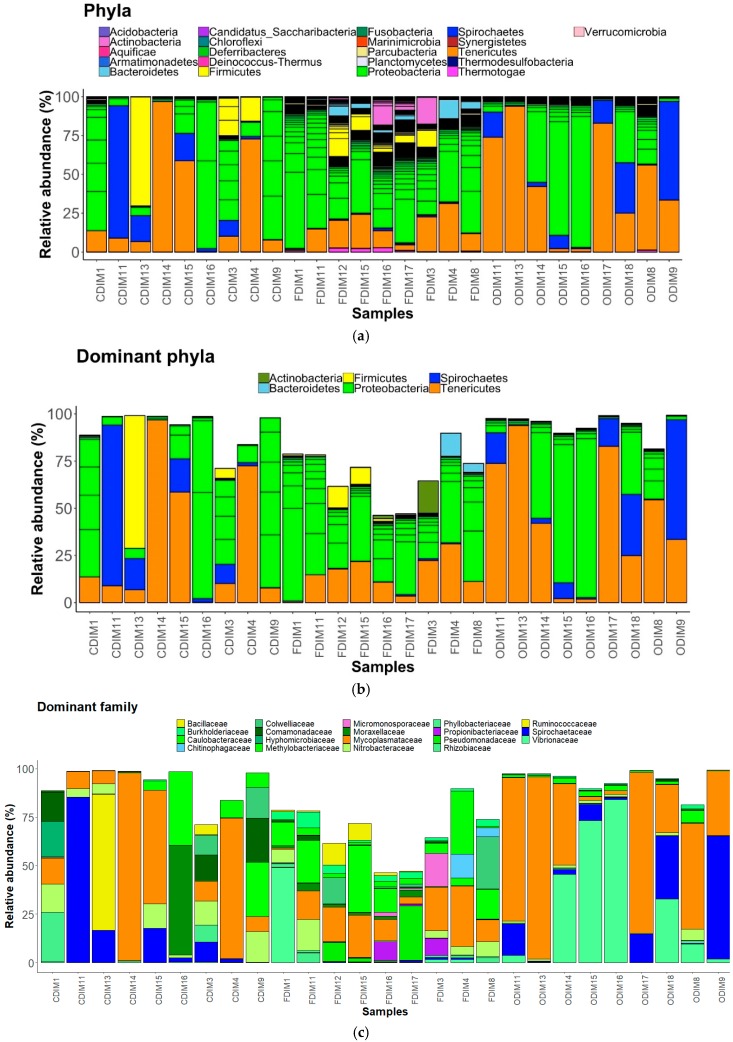
The relative abundance of the bacterial taxa present in the distal intestine mucus of Atlantic salmon. All the bacterial phyla (**a**), dominant phyla (**b**), and dominant families (**c**). Color codes for Proteobacteria—Shades of green, Spirochaetes—Dark blue, Firmicutes—Yellow, Actinobacteria—Orchid, and Tenericutes—Dark orange. The samples with C prefixes come from the control group. Similarly, those with F and O show the florfenicol- and oxolinic-acid-fed groups, respectively.

**Figure 4:**
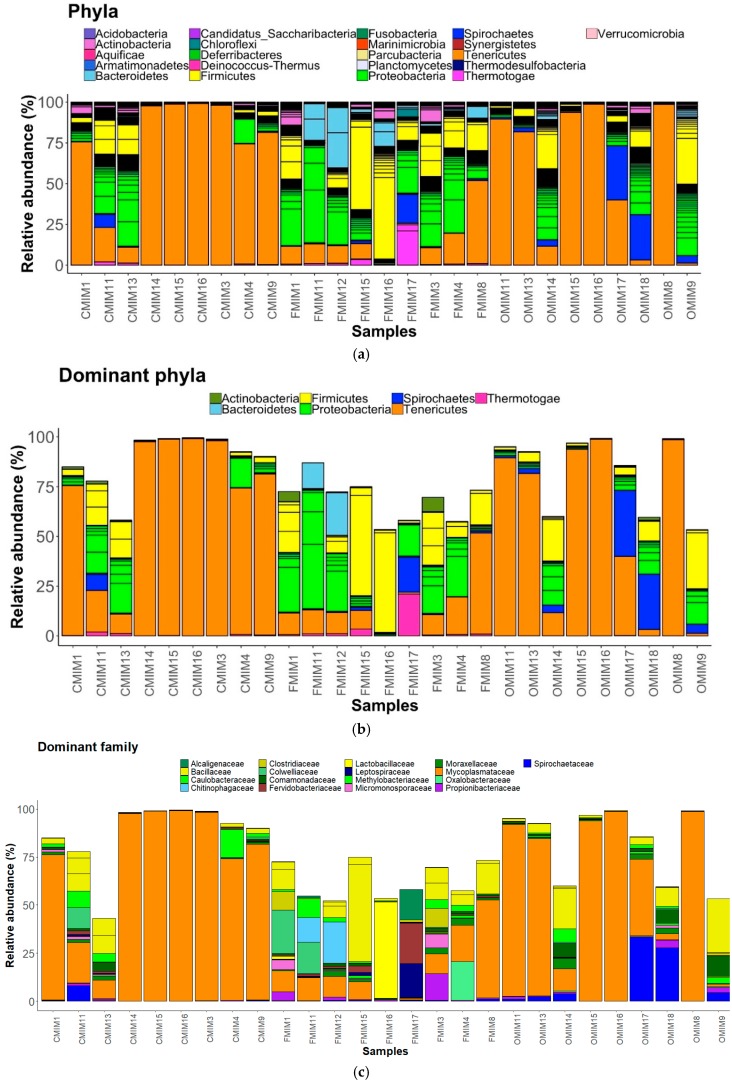
The relative abundance of the bacterial taxa present in the mid intestine mucus of Atlantic salmon. All the bacterial phyla (**a**), dominant phyla (**b**), and dominant families (**c**) in the mid intestine mucus of Atlantic salmon from the three fish groups. Color codes for Proteobacteria—Shades of green, Spirochaetes—Dark blue, Firmicutes—Yellow, Actinobacteria—Orchid, and Tenericutes—Dark orange. The samples with C prefixes come from the control group. Similarly, those with F and O show the florfenicol- and oxolinic-acid-fed groups, respectively.

We observed 21 phyla in the DI and MI (Figure 3A and Figure 4A). Proteobacteria, Actinobacteria, Firmicutes, Bacteroidetes, Spirochaetes, and Tenericutes were found to be dominant in the three study groups. However, Thermotogae was also found to be a dominant phylum in the MI of the three study groups (Figure 3B and Figure 4B). The average relative abundance (%) of the bacterial taxa is given in Table 2.

#### 3.3.1. DI Mucus

Phylum level: FFC feeding caused an increase in abundance of Actinobacteria, Proteobacteria, and Bacteroidetes, but decreased the abundance of Tenericutes, Spirochaetes, and Firmicutes compared to the control group. Proteobacteria were found to be more abundant than the rest (Figure 3A). OA feeding caused a general decrease in the abundance of Actinobacteria, Proteobacteria, and Firmicutes, but an increase in abundance of Tenericutes and Spirochaetes compared to the control group.

Family level: The families Micromonosporaceae and Propionibacteriaceae (Actinobacteria); Colwelliaceae, Comamonadaceae, Hyphomicrobiaceae, Methylobacteriaceae, Moraxellaceae, Phyllobacteriaceae, Pseudomonadaceae, Rhizobiaceae, Nitrobacteraceae, Burkholderiaceae, Caulobacteraceae, and Vibrionaceae (Proteobacteria); Chitinophagaceae (Bacteriodetes); Ruminococcaceae and Bacillaceae (Firmicutes); Mycoplasmtaceae (Tenericutes); and lastly, Spirochaetaceae (Spirochaetes) were found to be the dominant ones (Figure 3C). The family Mycoplasmataceae and the families belonging to Proteobacteria were found to be dominant than the rest in the three study groups. The abundance of Comamonadaceae and Moraxellaceae was found to be decreased, while that of Vibrionaceae increased in the antibiotic-fed fish compared to the control fish. The abundances of Mycoplasmataceae and Spirochaetaceae were found to be decreased in the F-fed group compared to the control.

Pearson’s Chi-squared test indicated that the proportions of the dominant bacterial taxa in the DI mucus of the three groups were significantly different for both bacterial phyla (χ^2^ = 58508, *p* < 0.05) and bacterial families (χ^2^ = 141300, *p* < 0.05).

#### 3.3.2. MI Mucus

Phylum level: The abundances of all the dominant phyla except Tenericutes were increased in the F-fed group compared to the control (Figure 4A,B). Tenericutes were found to be more abundant than the other phyla in the control and O-fed group (Figure 4A,B). The abundance of Thermotogae in OA-fed fish was lower, and those of Actinobacteria, Bacteroidetes, Firmicutes, and Spirochaetes were higher compared to the control fish.

Family level: Bacteria belonging to 19 families were present in the DI, and 17 families were present in the MI (Figure 3C and Figure 4C). The families Caulobacteraceae, Alcaligenaceae, Comamonadaceae, Colwelliaceae, Methylobacteriaceae, Moraxellaceae, and Oxalobacteraceae (Proteobacteria); Clostridiaceae, Bacillaceae and Lactobacillaceae (Firmicutes); Micromonosporaceae and Propionibacteriaceae (Actinobacteria); Chitinophagaceae (Bacteroidetes); Leptospiraceae, and Spirochaetaceae (Spirochaetes); Mycoplasmataceae (Tenericutes); and lastly Fervidobacteriaceae (Thermotogae) were found to be the dominant ones (Figure 4C). The abundance of Methylobacteriaceae and Mycoplasmataceae decreased in the antibiotic-fed groups compared to the control group. In contrast, the abundance of Comamonadaceae and Spirochaetaceae increased in the O-fed groups compared to the control group. Moraxellaceae was found to increase in the antibiotic-fed groups.

Pearson’s Chi-squared test indicated that the proportions of the dominant bacterial taxa in the MI mucus of the three groups were significantly different for both bacterial phyla (χ^2^ = 83049, *p* < 0.05) and bacterial families (χ^2^ = 98426, *p* < 0.05).

### 3.4. Core Bacterial Communities of the Intestinal Mucus Microbiota

**Figure 5:**
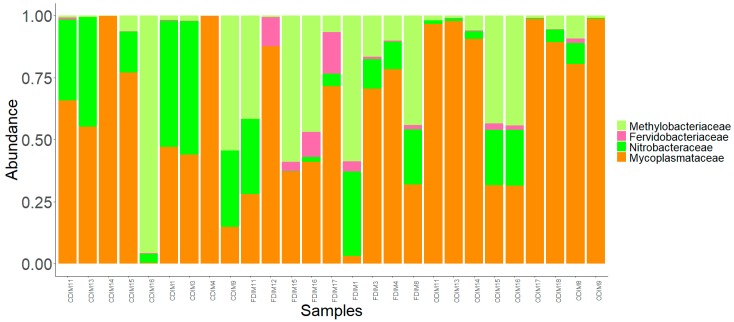
The core bacterial family in the distal intestine mucus of Atlantic salmon from the three fish groups. Color codes: shades of green—families of Proteobacteria, and dark orange—families of Tenericutes.

**Figure 6:**
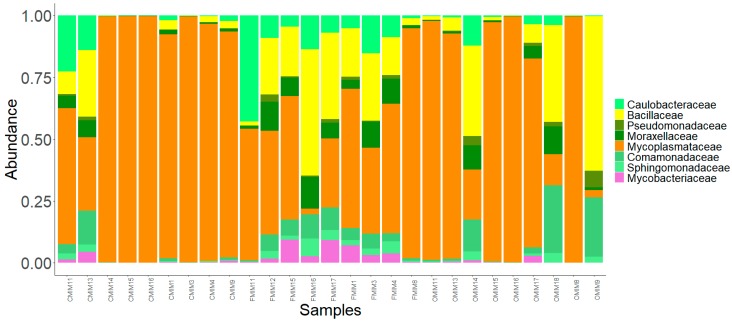
The core bacterial family in the mid intestine mucus of the three fish groups. Color codes: shades of green—families of Proteobacteria, yellow—families of Firmicutes, purple—families of Actinobacteria, and dark orange—families of Tenericutes.

In the present study, the core microbiota was identified as the members of the bacterial communities that were shared among 99% of the samples. The common core taxa—at a prevalence (relative population frequency) of 99% and compositional abundance detection threshold of 20%—are shown in Figure 5 and Figure 6. In the DI, only a few dominant bacterial families, namely Nitrobacteraceae, Mycoplasmataceae, and Methylobacteriaceae, were detected as the core members. Along with these dominant families, another family, Fervidobacteriaceae, was a member of the shared taxa in the DI (Figure 5). In the MI, the dominant bacterial families in the three study groups, namely Mycoplasmataceae, Comamonadaceae, Bacillaceae, Moraxellaceae, and Caulobacteraceae, were among the core members. In addition, Sphingomonadaceae, Mycobacteriaceae, and Pseudomonadaceae were also the core members of the MI in the three study groups (Figure 6).

The DPCoA indicated differences in the core members of the antibiotic-fed and the control group (DI mucus: F-statistic = 2.13, R^2^ = 0.15, *p* = 0.10; MI mucus: F-statistic = 3.42, R^2^ = 0.22, *p* = 0.04, Figure S3A,B).

### 3.5. Significantly Abundant Taxa of the Intestinal Mucus Microbiota

ANCOM analysis detected seven significantly abundant OTUs (compared to those in the control fish) in the DI, which included Cytophageaceae, Burkholderiaceae, Rhizobiaceae, two OTUs belonging to Sphingomonadaceae, and two OTU’s belonging to Flavobacteriaceae. ANCOM analysis did not detect any OTU that had significantly different abundances in the MI of the three groups.

### 3.6. Co-Occurrence Network Description of OTUs

A single-domain bacterial (SDB) network shows the co-occurrence networks to understand the possible biological interactions occurring among microbial communities. A typical SDB network consists of a set of nodes and a set of edges. The nodes indicate OTUs or phyla, and edges indicate the line connecting the two nodes. The number of connections that one node has with other nodes is the degree of a node. The number of the shortest path that passes through the nodes in the network is evaluated as betweenness. Furthermore, a selectively connected labeled pair of nodes is quantified by the assortativity coefficient.

#### 3.6.1. DI Mucus Bacteria

**Figure 7:**
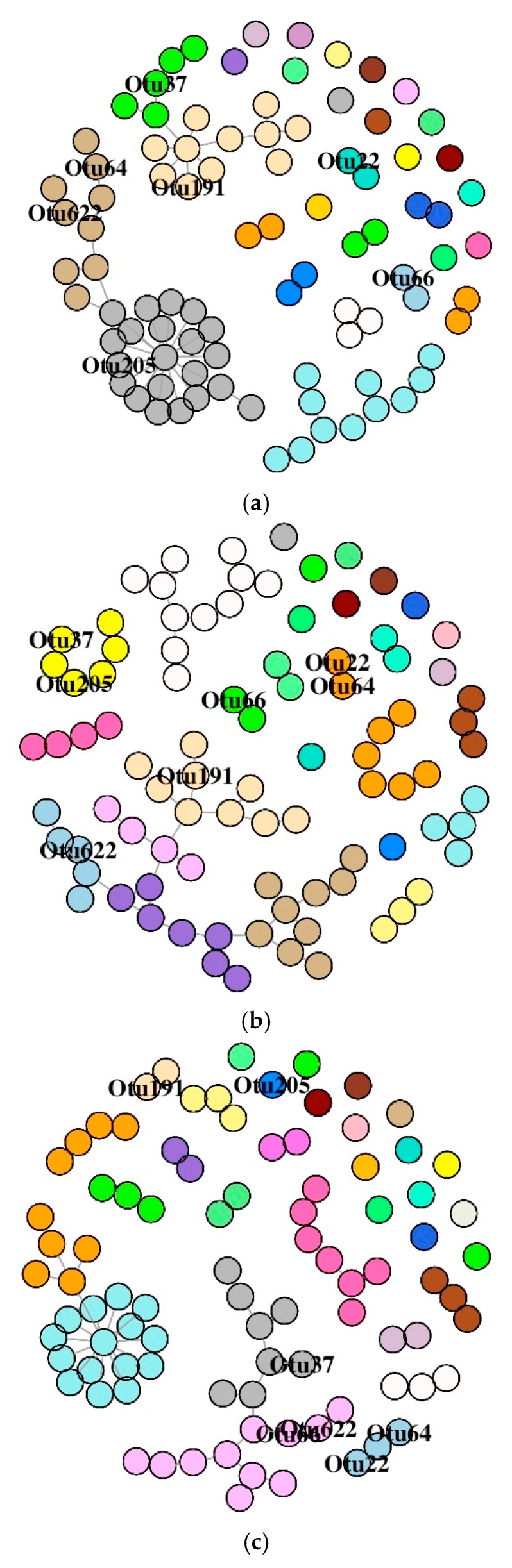
Association network graphs of the significantly abundant OTUs of Atlantic salmon. Distal intestine mucus bacterial networks of the control (**a**), florfenicol-fed (**b**), and oxolinic-acid-fed (**c**) fish. Nodes represent OTUs and specific colors of the modules reveal the memberships of the significantly abundant OTUs.

The SDB network associated with the DI of the three study groups is comprised of many small components (Figure S4A). Labelling of the significantly abundant OTUs based on their membership in different modules (Figure 7A–C) revealed their differential positioning in the three study groups. We observed that the number of dyads, triads, quadruplets, and quintuplets in bacterial networks had no connection with the main connected network; their connection patterns were different for all the three study groups (Figure S4A). The average node degrees were 1.49 (SD: 2.17), 1.56 (SD: 1.02), and 1.36 (SD: 1.68) for the control, F-, and O-fed fish, respectively. Similarly, the values for betweenness were 14.8 (SD: 46.1), 30.3 (SD: 69.4), and 9.03 (SD: 25.5) for the control, F-, and O-fed fish, respectively. The average path lengths of the SDB network for the three study groups (control, F-fed, and O-fed) were 3.14, 5.07, and 2.99. The average node degrees and betweenness of the three groups were found to be different (for node degree: *p* = 0.12 for F-fed vs. control group and *p* = 0.06 for O- vs. F-fed group; for betweenness: *p* = 0.03 for F-fed vs. control group and *p* = 0.03 for O- vs. F-fed group). The degrees of assortativity (assortativity coefficient *c_a_*) of the network associated with the three groups were −0.27, 0.07, and −0.23, respectively. While there were two distinct groups of nodes in the control and O-fed groups, there were four distinct groups of nodes in the F-fed group (Figure S5A). The control and O-fed group had some highly connected hub nodes in the bacterial network, and the hubs of the control group had more node degrees. 

#### 3.6.2. MI Mucus Bacteria

**Figure 8:**
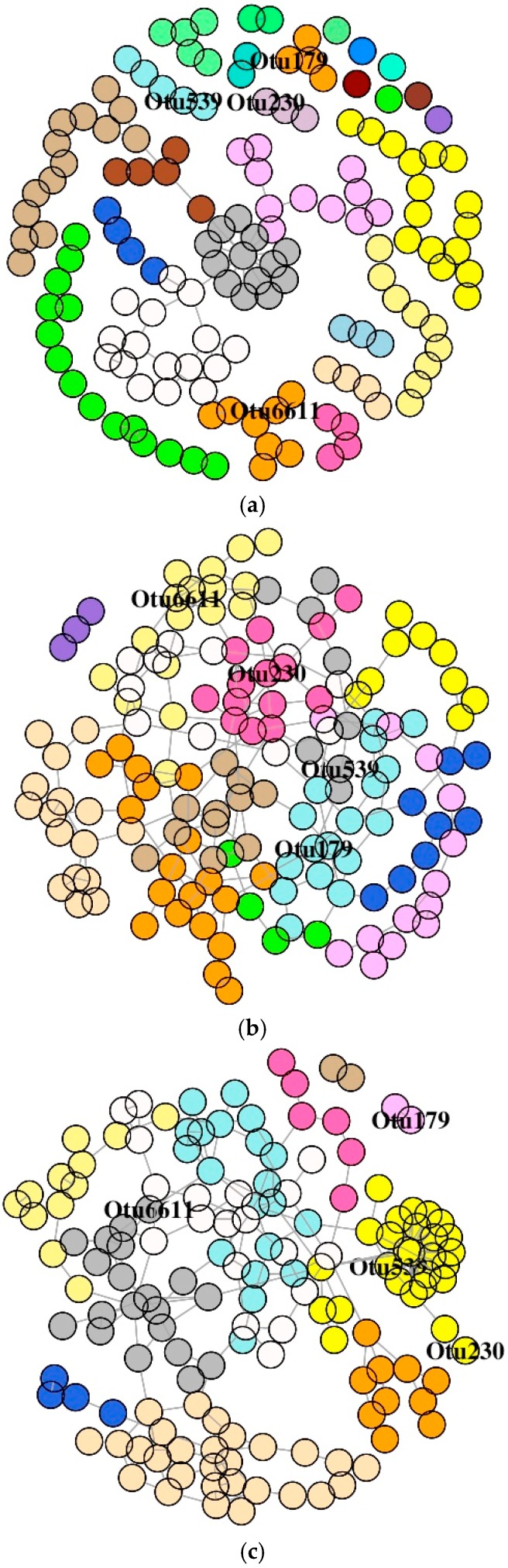
Association network graphs of the significantly abundant OTUs of Atlantic salmon. Mid intestine mucus bacterial networks of the control (**a**), florfenicol-fed (**b**), and oxolinic-acid-fed (**c**) fish. Nodes represent OTUs and specific colors of the modules reveal the memberships of the significantly abundant OTUs.

The SDB network derived from the MI of the antibiotic-treated groups was comprised of one giant connected component (Figure 8A–C). The SDB of the control group had small components. Similar to the observation of the DI bacterial network, the dyads, triads, quadruplets, and quintuplets in the MI bacterial networks of three groups were also different (Figure S4B). The average node degrees were 1.85 (SD: 1.49), 2.76 (SD: 1.17), and 3.03 (SD: 2.31) for the control, F-, and O-fed fish, respectively. Similarly, the values for betweenness were 247 (SD: 520), 404 (SD: 375), and 393 (SD: 599) for the control, F-, and O-fed fish, respectively. The average path lengths of the SDB network for the three study groups (control, F-fed, and O-fed) were 8.86, 6.64, and 6.57, respectively. Dunn’s test identified significant differences between the antibiotic-fed groups and the control group (for average node degree: *p* < 0.001 for both control vs. F-fed group and control vs. O-fed group; for betweenness: *p* < 0.001 for both control vs. F-fed group and control vs. O-fed group, *p* = 0.02 for F-fed vs. O-fed group). The degree of assortativity (assortativity coefficient *c_a_*) of the phylum-level network of the three groups was −0.17, 0.04, and −0.13, respectively. The histogram showing the node degree distribution of the microbial networks (for all OTUs) of the three study groups (Figure S5B) revealed that there were two distinct groups of nodes in the control and O-fed group, while there were four distinct groups of nodes in the F-fed group. Visualization of this network also showed that hubs of the control and O-fed group had more node degrees. 

The main results of this study are summarized in Supplementary Figure S6.

## 4. Discussion

Antibiotics are antimicrobial agents that are employed to treat infections. Intake of antibiotics can selectively deplete the gut microbial populations of the host, depending on their mode of action [43]. Furthermore, they will affect not only the targeted microbes, but also the host’s entire microbial community [44]. In the present study, we employed amplicon sequencing of highly conserved 16S rRNA gene sequences to investigate changes in the intestinal microbiota of Atlantic salmon after feeding them with one of the two antibiotics, namely florfenicol and oxolinic acid. In the present study, we focused on the intestinal mucus of the host because it consists of a unique microbial niche with distinct communities that have a special functional role in the host–microbial relationship [45]. The microbial niche of the intestinal mucus layer provides partial protection against several pathobionts and opportunistic microbes, the presence of which can cause mucosal infections [46]. 

Along with alterations in the diversity and composition of the intestinal microbes, the co-occurrence patterns were also altered after the antibiotic feeding. In addition, we confirmed that the tank biofilm bacterial communities might not have influenced the intestinal bacterial profile, as reported earlier [29].

### 4.1. Antibiotic Feeding Lifted the Richness and Diversity of the Intestinal Microbes

The observed increase in alpha diversity in the present research was not consistent with the significant decrease in the diversity reported by previous antibiotic studies with mice, humans, and fish [47,48,49]. However, a recent study has indicated an increase in the bacterial diversity (richness and Shannon diversity index) in fecal samples of minks that were collected 2 days after oral administration of amoxicillin [50]. According to the intermediate disturbance hypothesis theory, the bacterial population maximizes its diversity at intermediate rates of disturbance [51]. Intermediate levels of antibiotics are associated with increases in the diversity of bacterial colony size phenotype [52]. This disturbance–diversity relationship depends on the colonizing (favored by r-selection) and competitive (favored by k-selection) ability of the bacterial population under different rates/levels of disturbances. At intermediate levels of disturbance, coexistence of microorganisms that can thrive in different environments causes peaks in diversity, resulting in a unimodal disturbance–diversity relationship [51]. The medicated feeds had different levels of antibiotics, and we did not observe significant changes in diversity in O-fed fish (in MI) similar to those noted for F-fed fish. By linking this finding and the intermediate disturbance hypothesis, we suggest that an intermediate level of disturbance was induced in F-fed fish to cause the co-existence of both r- and k-strategists.

### 4.2. Antibiotic Feeding Altered the Composition of the Intestinal Mucus Microbial Consortia

Administration of antimicrobial agents can dramatically disturb the ecological balance between the host and its associated microorganisms. The mode of action of the bacteriostatic antibiotics is via suppressing bacterial growth, mostly through inhibiting the process of protein synthesis and interfering with bacterial replication [25,53]. Moreover, broad-spectrum antimicrobials such as those employed in the present study are effective against a wide range of commensal and pathogenic bacteria. The mode of action of the antibiotic has a bearing on the extent to which the gut microbiota composition and functions are modulated in humans [53]. Our findings demonstrate that consumption of antibiotics shifted the intestinal microbiota composition in salmon. The composition and abundance of the dominant bacterial phyla, namely Proteobacteria, Actinobacteria, Firmicutes, Spirochaetes, Bacteroidetes, Tenericutes, and Thermotogae, were altered in the distal and mid intestine of antibiotic-fed fish compared to control fish (Figure 3B and Figure 4B). The bacterial families that were influenced due to antibiotic feeding are briefly discussed in the following paragraphs. 

Antibiotic feeding caused a general increase in abundance of the phylum Proteobacteria in intestinal mucus (Figure 3A and Figure 4A). Furthermore, the significantly abundant families that were detected in the DI belonged to Proteobacteria and Bacteroidetes. Similar to our observations, studies on F-fed channel catfish and mice have also reported an increase in the abundance of Proteobacteria and Bacteroidetes [47,49]. However, the phylum Proteobacteria was low in abundance in the DI of O-fed group compared to control group. Members of this phylum are abundant in many marine and freshwater fishes [54], and are also known to dominate the gut microbiota of Atlantic salmon [55,56]. Previous studies have shown that Proteobacteria contributes to the digestive process in fish [57]. Members of this phylum are also involved in the stress response regulatory system and in metabolic pathway modules that participate in carbon and nitrogen fixation [58]. While most members of Proteobacteria had a higher abundance in the antibiotic-fed groups, there were also members that had a low abundance in the antibiotic-fed fish. Burkholderiaceae, Rhizobiaceae, and Sphingomonadaceae were significantly abundant in the DI of F-fed fish. Although Vibrionaceae was not a significantly abundant OTU, its abundance was higher in the O-fed fish. The members of the genus *Vibrio* are known to be opportunistic pathogens in fish [59,60], and these r-strategists were higher in the microbiota of black molly (*Poecilia sphenops*) exposed to streptomycin [61].

While two families within Actinobacteria had higher abundance only in the DI of the F-fed fish compared to the control fish, the abundances of two families within Firmicutes were mostly increased by antibiotic feeding. One of these families, Bacillaceae, was detected as a member of the core microbiota in the MI (Figure 6). Members belonging to Bacillaceae include both free-living and pathogenic species [62], for example, *Bacillus mycoides* are considered pathogenic to some fish species [63].

Two members of Spirochaetes—Spirochaetaceae and Leptospiraceae—displayed antibiotic-specific alterations (Figure 3C and Figure 4C). Within the family Leptospiraceae, *Leptospira* is known to cause Leptospirosis in humans and animals [64]. A high prevalence of *Leptospira* in catfish and tilapia species has been previously reported [65]. The finding on differential abundance patterns of families belonging to Spirochaetes merits further investigation. The functional importance and pathogenicity of these bacteria in salmon need to be elucidated.

It was interesting to find a general decrease in abundance of Mycoplasmataceae (Tenericutes), the dominant family in Atlantic salmon intestine [29,56,66,67], in the antibiotic-fed groups compared to the control group. It should be noted that Mycoplasmataceae was also a core member of the intestinal microbiota of salmon. Further, the phylum Thermotoage (family Fervidobacteriaceae) was found to be a dominant member only in the MI of the fish. We observed a general increase in the abundance of Fervidobacteriaceae in the antibiotic-fed groups. The presence of Thermotoage in the salmon intestine has been previously reported [56].

### 4.3. Antibiotics Affected the Intestinal Mucus Microbial Association and Stability

Inferring interactions among different microbes within a community is vital to understanding how the microbes adapt, develop, and interact with the host [29]. The diverse microbes residing in the intestine interact with each other to obtain nutrients required for their colonization and proliferation, and these interactions occur by developing complex ecological networks i.e., microbe–microbe associations. Such microbial networks help to establish intestinal microbial compositional stability [68]. Exposure to antibiotics can collapse this stability and thereby disturb the interactions among microbial species. We inferred single-domain networks using the SPEIC-EASI framework.

The inferred SDB network of the DI and MI bacteria of F-fed fish had higher overall connectivity, betweenness, and hubs with more node degree (Figure S5A,B). On the other hand, inferred SDB network for the O-fed fish (DI) had lower overall connectivity, betweenness, and hubs with lesser node degree. The higher selective linking and higher average node degree of the F-fed group indicate more interactions among the gut bacteria, probably an indication of cooperative microbial communities that are functionally dependent. An increase in microbial diversity along with a higher proportion of cooperative microbial interactions can disturb stability [69]. Higher cooperation among the microbes leads to over-representation of the most stable communities, which in turn can cause a runaway effect that can disintegrate the competing microbial population [70].

In the DI of the antibiotic-fed fish, most of the labeled OTUs belonged to different modules. However, Rhizobiaceae was connected to other significantly different OTUs, and members of this family are known for their ability to establish a beneficial interaction with the host (plants) and participate in the process of biological nitrogen fixation [71]. In the MI, the labeled OTUs in the F-fed group were connected to the main network. However, they belonged to different modules. In the O-fed group, the labeled OTUs were connected to the main network and belonged to different modules, except one OTU that belonged to the phylum Proteobacteria (family Halomonadaceae), which was in a dyad. This suggests that both FFC and OA can differentially affect microbe–microbe interactions (Figure S4A,B). The alteration of the membership in the predicted network after antibiotic feeding has to be further investigated.

## 5. Conclusions

In conclusion, antibiotic exposure increased the bacterial diversity of the distal intestine and shifted the intestinal bacterial community composition in Atlantic salmon. Florfenicol feeding caused the intestinal microbial communities to be more diverse compared to the other study groups. Antibiotic feeding altered the composition and abundance of the dominant bacterial phyla, namely Proteobacteria, Actinobacteria, Firmicutes, Spirochaetes, Bacteroidetes, Tenericutes, and Thermotogae. Certain families that were low in abundance in control fish became abundant in fish that consumed medicated feed. Furthermore, the two antibiotics even disturbed the core microbiota of the fish. The co-occurrence networks of the intestinal bacteria indicated that the antibiotics affected the microbe–microbe interactions differentially. Though intriguing, the results improve our understanding of the structure, diversity, and composition of the Atlantic salmon intestinal microbiota following antibiotic intervention.

## Figures and Tables

**Table 1:** Species richness and Shannon and Simpson diversity of bacterial communities in the control and antibiotic fed groups.

Alpha Diversity	Distal Intestine				Mid Intestine			
	Groups	*p*-Value	Groups	Mean ± SD	Groups	*p*-Value	Groups	Mean ± SD
Species richness	FDM-CDM	0.000	CDM	55.11 ± 19.23 ^a^	FMM-CMM	0.223	CMM	120.78 ± 59.28 ^a,b^
	ODM-CDM	0.104	FDM	215.22 ± 59.11 ^b^	OMM-CMM	1.000	FMM	168.44 ± 37.71 ^b^
	ODM-FDM	0.130	ODM	120.67 ± 55.65 ^a,b^	OMM-FMM	0.048	OMM	109.44 ± 38.31 ^a^
Shannon diversity	FDM-CDM	0.001	CDM	4.51 ± 3.52 ^a^	FMM-CMM	1.121	CMM	1.22 ± 1.28
	ODM-CDM	1.000	FDM	21.38 ± 12.22 ^b^	OMM-CMM	1.000	FMM	2.68 ± 0.43
	ODM-FDM	0.001	ODM	3.68 ± 2.36 ^a,b^	OMM-FMM	0.462	OMM	1.62 ± 1.41
Simpson diversity	FDM-CDM	0.020	CDM	3.61 ± 3.06 ^a^	FMM-CMM	0.108	CMM	3.80 ± 4.99
	ODM-CDM	1.000	FDM	8.90 ± 4.19 ^b^	OMM-CMM	1.000	FMM	7.48 ± 3.77
	ODM-FDM	0.001	ODM	2.10 ± 0.85 ^a,b^	OMM-FMM	0.297	OMM	4.69 ± 4.80
PD	FDM-CDM	0.001	CDM	183.05 ± 46.57 ^a^	FMM-CMM	0.112	CMM	312.29 ± 108.90 ^a^
	ODM-CDM	0.121	FDM	478.74 ± 99.51 ^b^	OMM-CMM	1.000	FMM	400.98 ± 57.80 ^a,b^
	ODM-FDM	0.121	ODM	319.22 ± 105.72 ^a,b^	OMM-FMM	0.066	OMM	291.83 ± 72.38 ^a,c^

Statistically significant differences (*p* < 0.05) of a particular diversity measure are shown using different letters.

**Table 2:** Average relative abundance (%) of the dominant intestinal bacteria.

Groups	Control		F-Fed Group		O-Fed Group	
Sample Type	DI	MI	DI	MI	DI	MI
**Phyla**						
Proteobacteria	41.64 ± 40.32	14.99 ± 19.44	61.01 ± 17.98	33.71 ± 18.32	37.23 ± 36.10	17.75 ± 19.26
Bacteroidetes	0.06 ± 0.05	0.98 ± 0.97	5.73 ± 4.23	11.04 ± 12.66	0.55 ± 0.54	2.23 ± 2.57
Tenericutes	30.55 ± 35.59	72.78 ± 34.25	14.91 ± 9.58	13.80 ± 14.97	45.48 ± 33.53	57.63 ± 43.13
Firmicutes	12.45 ± 23.38	6.87 ± 9.91	10.05 ± 7.95	28.86 ± 22.03	0.67 ± 0.86	11.73 ± 14.71
Actinobacteria	0.25 ± 0.38	2.41 ± 2.71	5.62 ± 7.37	5.16 ± 5.34	0.13 ± 0.15	2.28 ± 2.39
Spirochaetes	14.90 ± 27.33	1.20 ± 2.80	0.92 ± 0.53	2.74 ± 5.94	15.60 ± 20.83	8.17 ± 12.89
Thermotogae	-	0.54 ± 0.72	-	3.75 ± 7.96	-;	0.03 ± 0.05
**Family**						
Mycoplasmataceae	30.55 ± 35.59	72.78 ± 34.26	14.92 ± 9.57	13.80 ± 14.97	45.48 ± 33.52	57.63 ± 43.13
Comamonadaceae	5.77 ± 8.97	2.95 ± 7.04	1.35 ± 0.83	2.47 ± 4.28	0.19 ± 0.21	3.76 ± 4.34
Bacillaceae	0.71 ± 1.74	5.79 ± 8.75	4.87 ± 5.77	14.30 ± 16.75	0.05 ± 0.07	9.59 ± 12.02
Sphingomonadaceae	0.00 ± 0.01	0.51 ± 0.58	1.09 ± 1.11	0.85 ± 0.94	0.16 ± 0.17	0.86 ± 0.94
Moraxellaceae	6.39 ± 18.77	1.00 ± 1.06	2.17 ± 1.39	1.63 ± 1.42	0.24 ± 0.33	2.82 ± 3.67
Mycobacteriaceae	0.00 ± 0.00	0.40 ± 0.51	0.39 ± 0.40	0.73 ± 0.64	0.01 ± 0.27	0.35 ± 0.50
Caulobacteraceae	1.84 ± 3.66	1.90 ± 2.92	5.23 ± 10.30	2.50 ± 3.03	0.13 ± 0.11	1.26 ± 2.18
Pseudomonadaceae	0.26 ± 0.45	0.25 ± 0.29	6.00 ± 9.41	2.44 ± 2.06	0.19 ± 0.28	1.33 ± 1.54
Alcaligenaceae	0.00 ± 0.00	0.04 ± 0.11	0.15 ± 0.40	1.87 ± 5.18	0.00 ± 0.00	0.04 ± 0.12
Chitinophagaceae	0.00 ± 0.00	0.08 ± 0.15	1.88 ± 4.16	3.83 ± 7.85	0.02 ± 0.02	0.17 ± 0.46
Clostridiaceae	1.11 ± 3.33	0.19 ± 0.22	1.08 ± 1.75	3.22 ± 4.67	0.00 ± 0.00	0.36 ± 0.85
Colwelliaceae	2.95 ± 5.91	1.67 ± 4.49	4.78 ± 9.32	4.41 ± 8.68	0.01 ± 0.02	0.27 ± 0.36
Fervidobacteriaceae	0.07 ± 0.04	0.51 ± 0.70	1.16 ± 1.06	3.56 ± 7.58	0.19 ± 0.35	0.03 ± 0.05
Lactobacillaceae	0.01 ± 0.01	0.18 ± 0.17	0.83 ± 0.46	6.19 ± 16.51	0.29 ± 0.38	0.29 ± 0.43
Leptospiraceae	0.05 ± 0.06	0.22 ± 0.29	0.71 ± 0.59	3.46 ± 8.44	0.10 ± 0.16	0.01 ± 0.02
Methylobacteriaceae	8.00 ± 14.43	1.77 ± 4.90	11.77 ± 11.13	0.49 ± 0.48	2.15 ± 1.79	0.92 ± 1.13
Micromonosporaceae	0.03 ± 0.08	0.38 ± 0.54	2.25 ± 5.61	1.54 ± 2.66	0.00 ± 0.01	0.26 ± 0.48
Oxalobacteraceae	0.00 ± 0.00	0.13 ± 0.26	1.20 ± 2.56	2.27 ± 6.76	0.02 ± 0.04	0.24 ± 0.23
Propionibacteriaceae	0.00 ± 0.01	0.37 ± 0.44	2.26 ± 4.04	2.57 ± 4.52	0.02 ± 0.01	1.08 ± 1.32
Spirochaetaceae	14.88 ± 27.32	1.04 ± 2.64	0.51 ± 0.38	0.43 ± 0.35	15.56 ± 20.85	8.16 ± 12.89
Vibrionaceae	1.18 ± 2.36	ND	6.78 ± 17.12	ND	29.08 ± 34.57	ND

The values represent mean ± SD. ND: not dominant.

## Supplementary Materials

The following are available online at https://www.mdpi.com/2076-2607/7/8/233/s1. Figure S1: Sample-size-based rarefaction curves for the reads obtained from Atlantic salmon intestinal mucus—Distal (**A**), mid (**B**), and tank biofilm (**C**) samples. The shaded portion around each line represents the 95% confidence interval. Color codes for the distal intestine samples: green lines—Control group, red lines—Florfenicol-fed group, blue lines—Oxolinic-acid-fed group. Color codes for the mid intestine samples: grey lines—Control group, orange lines—Florfenicol-fed group, yellow lines—Oxolinic-acid-fed group. Color codes for the tank biofilm samples: orange lines—Control group, green lines—Florfenicol-fed group, purple lines—Oxolinic-acid-fed group. Figure S2: Double principal coordinate analysis plots showing the beta diversity of the bacterial communities of Atlantic salmon intestine and biofilm. Tank biofilm bacteria (**A**), control group distal intestine and tank biofilm bacteria (**B**): F-statistic = 4.035, R^2^ = 0.211, *p* = 0.01; F group distal intestine and tank biofilm bacteria (**C**): F-statistic = 2.375, R^2^ = 0.136, *p* = 0.07; O group distal intestine and tank biofilm bacteria (**D**): F-statistic = 5.006, R^2^ = 0.250, *P* = 0.002; control group mid intestine and tank biofilm bacteria (**E**): F-statistic = 16.291, R^2^ = 0.520, *p* = 0.003; F group mid intestine and tank biofilm bacteria (**F**): F-statistic = 2.934, R^2^ = 0.163, *p* = 0.051; O group mid intestine and tank biofilm bacteria (**G**): F-statistic = 3.910, R^2^ = 0.206, *p* = 0.03. Figure S3: Double principal coordinate analysis plots showing the differences in the composition of the core bacterial communities in the intestine mucus of Atlantic salmon. The data in the ellipse-enclosed areas come from a multivariate normal distribution. Distal intestine (**A**): F-statistic = 2.061, R^2^ = 0.120, *p* = 0.082 and mid intestine (**B**): F-statistic = 2.061, R^2^ = 0.120, *p* = 0.082 samples of the antibiotic-fed and control-fed groups. Figure S4: Single-domain network graph of the bacteria in the intestine mucus of Atlantic salmon. Distal intestine (4**A**) and mid intestine (4**B**) of the three fish groups. Phyla in different nodes are color-coded. The three panels represent the three feed groups: Control (**A**), F-fed (**B**), O-fed (**C**). Figure S5: Histograms showing the degree distribution of the bacterial networks associated with the intestine of Atlantic salmon. Distal intestine (5**A**) and mid intestine (5**B**). Figure S6: Summary of the main findings in the study. DI, distal intestine; MI, mid intestine; C, control group; F-fed and O-fed, antibiotic-fed groups.
