# Supplementary material for: Antibiotic-Induced Perturbations Are Manifested in the Dominant Intestinal Bacterial Phyla of Atlantic Salmon
Source: Microorganisms. 2019 Aug 2;7(8):233. doi: 10.3390/microorganisms7080233 (PMC6723382; doi:10.3390/microorganisms7080233)

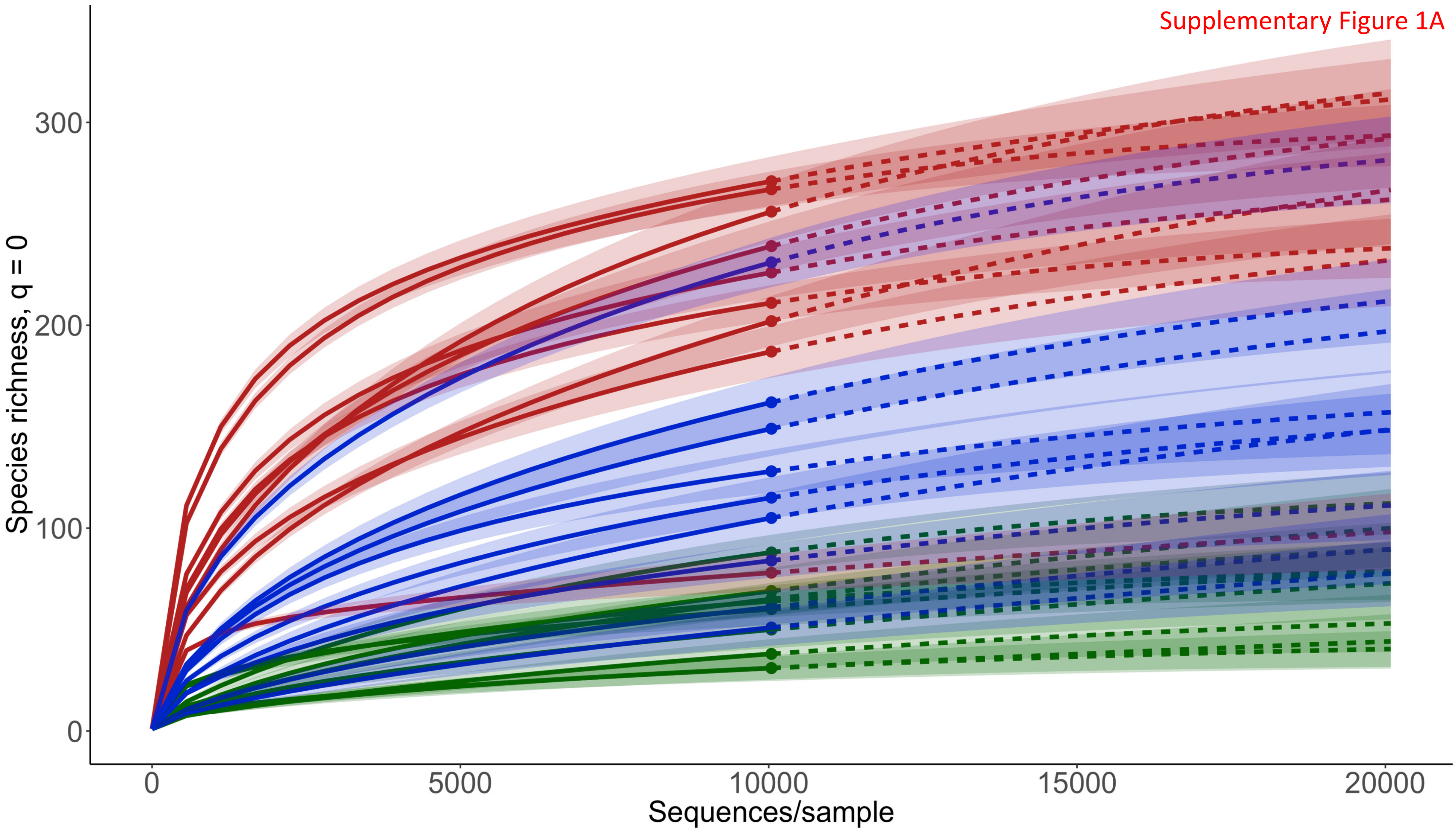

Supplementary Figure 1A
Species richness, q = 0
300
200
100
0
0
5000
10000
15000
20000
Sequences/sample

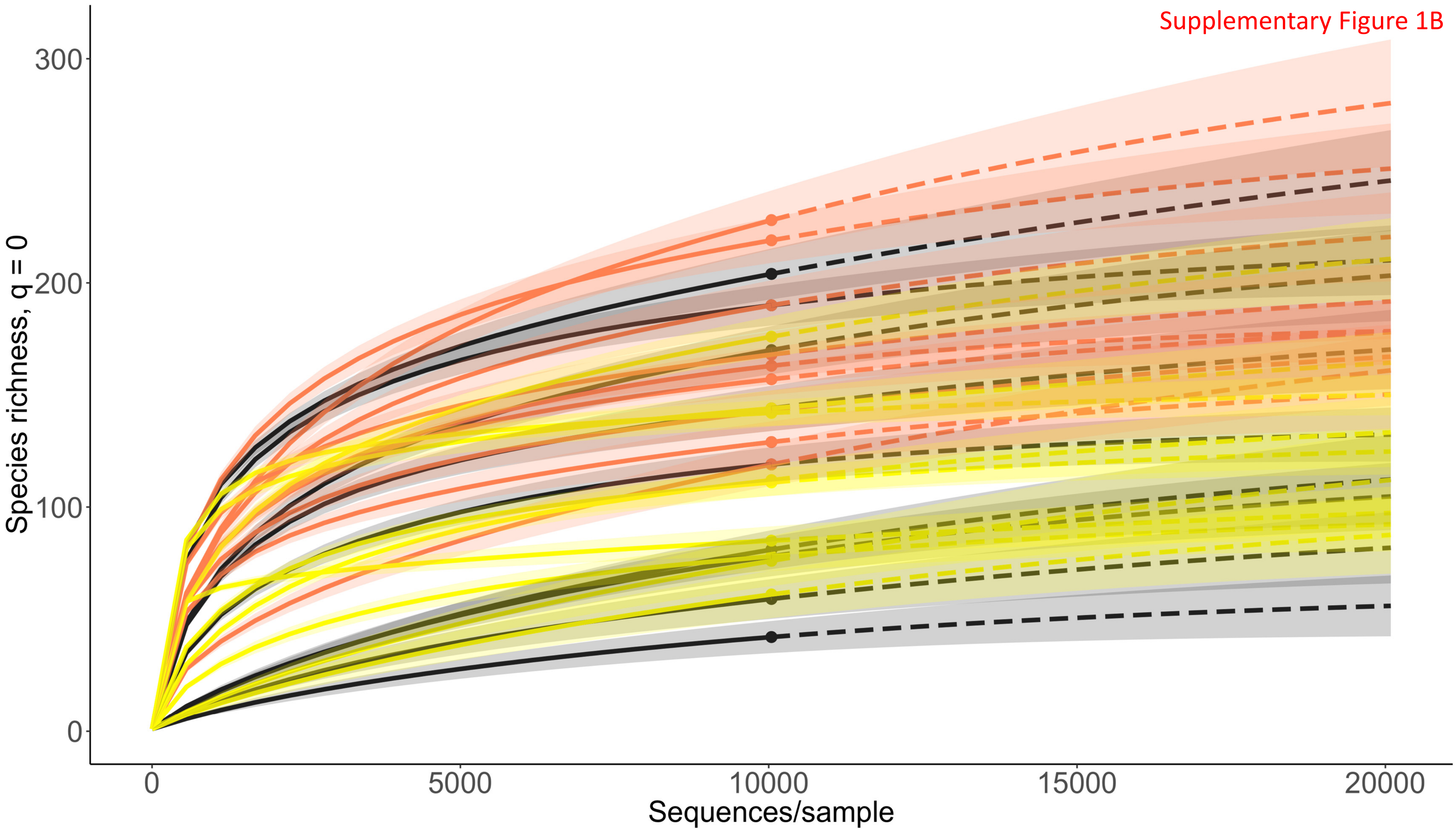
Supplementary Figure 1B
Species richness, q = 0
0
100
200
300
0
5000
10000
15000
20000
Sequences/sample

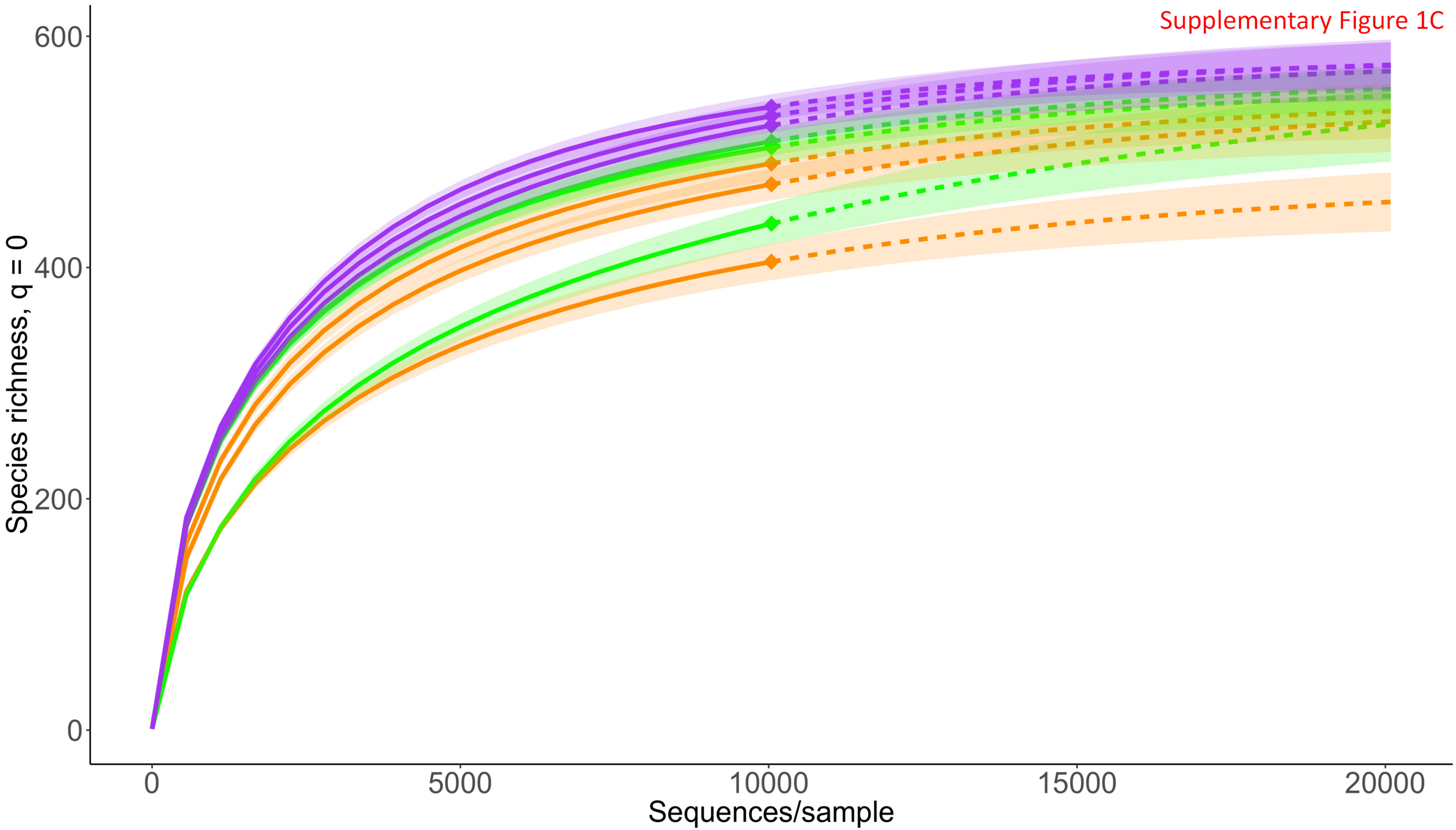
Supplementary Figure 1C
600
400
200
0
Species richness, q = 0
0
5000
10000
15000
20000
Sequences/sample

Supplementary Figure 2A

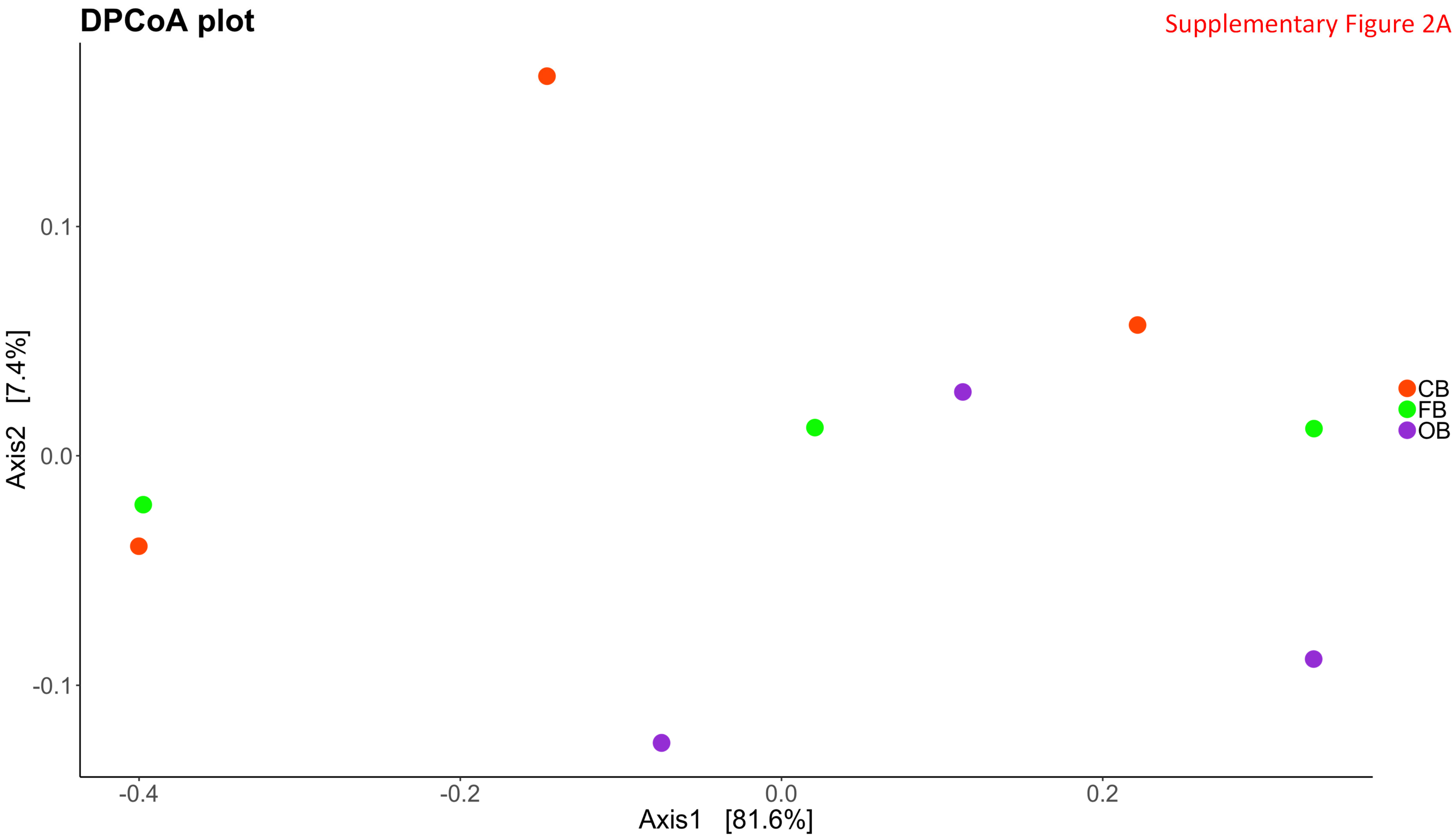

Supplementary Figure 2B

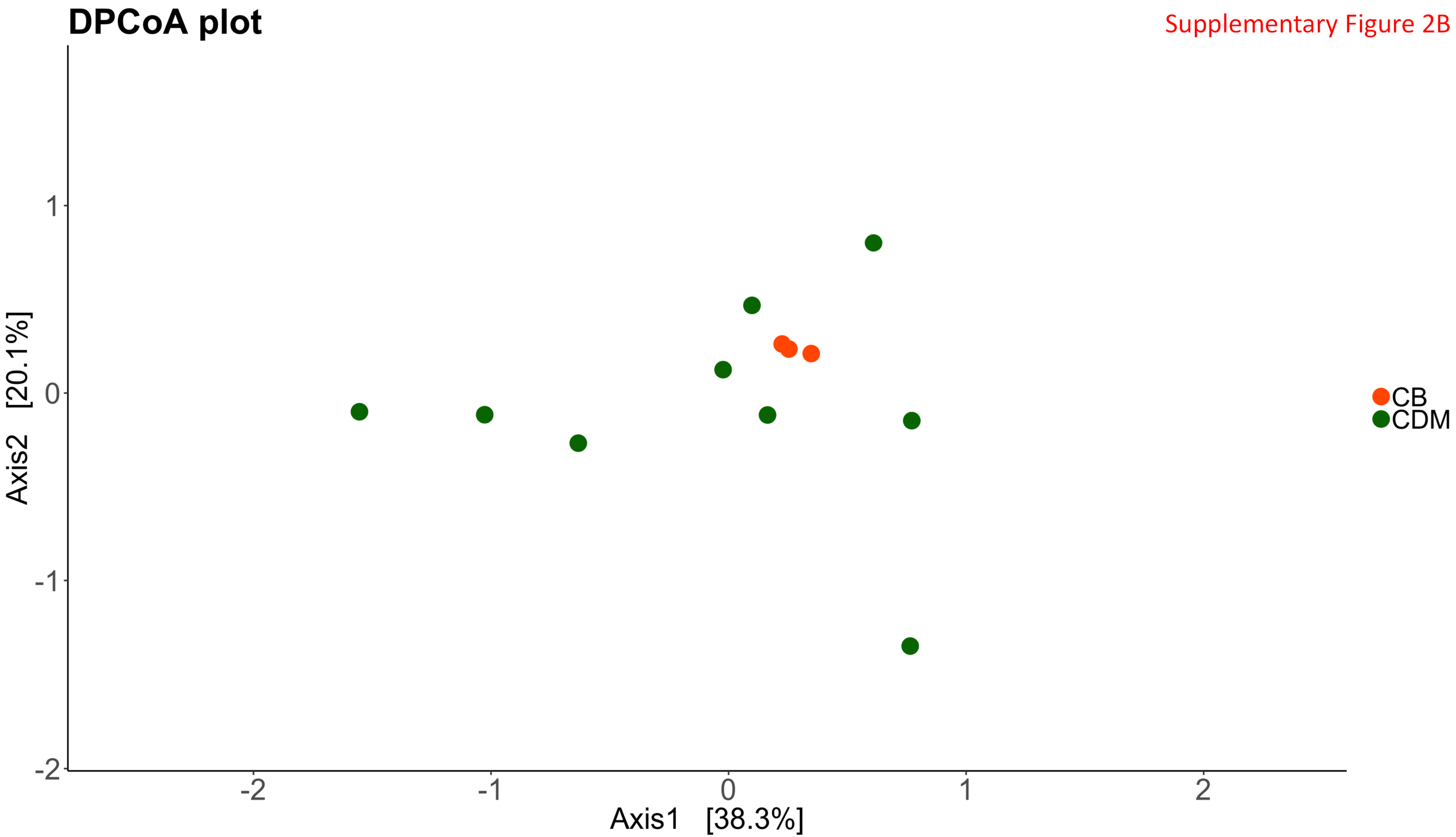

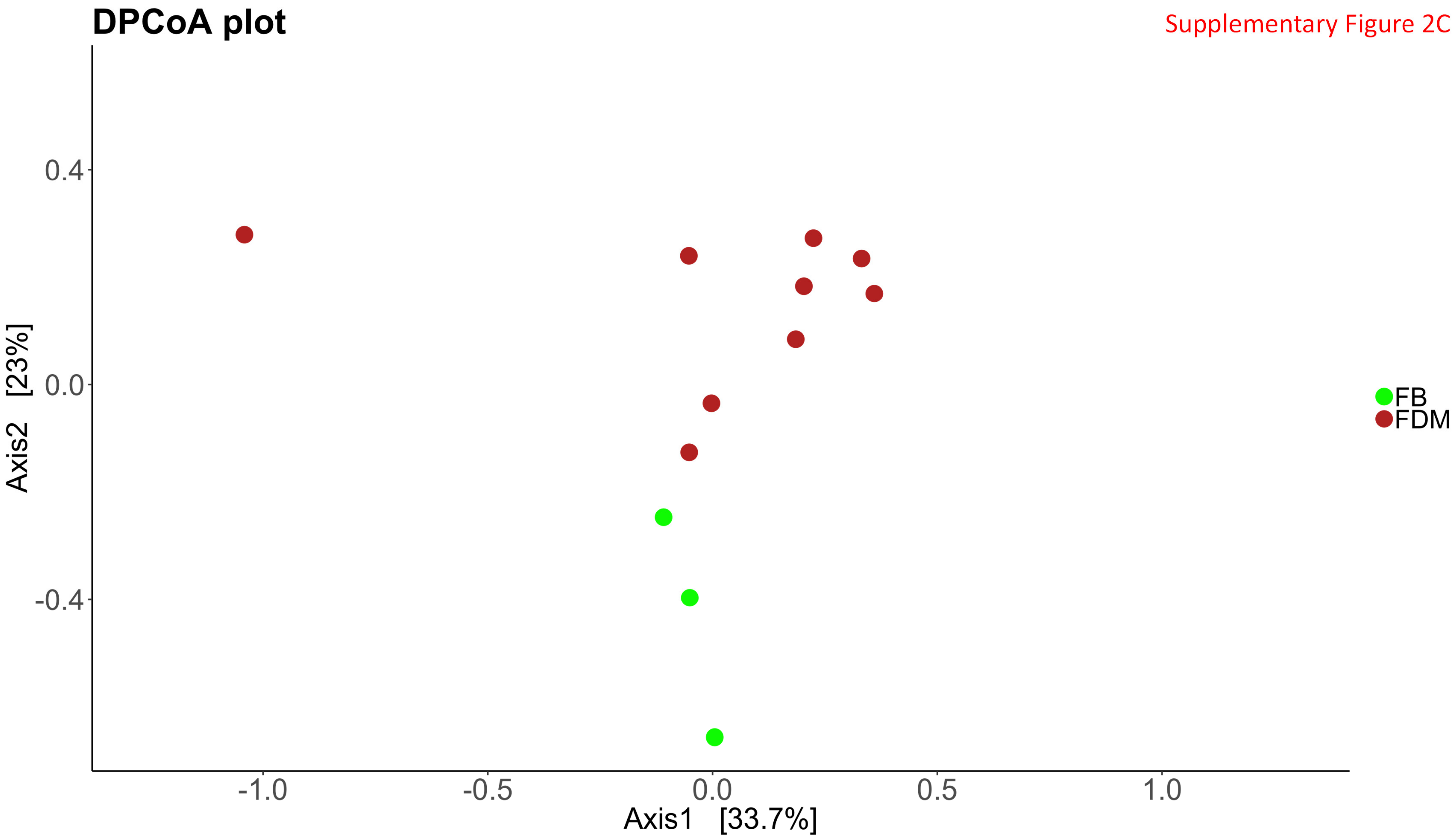

Supplementary Figure 2C

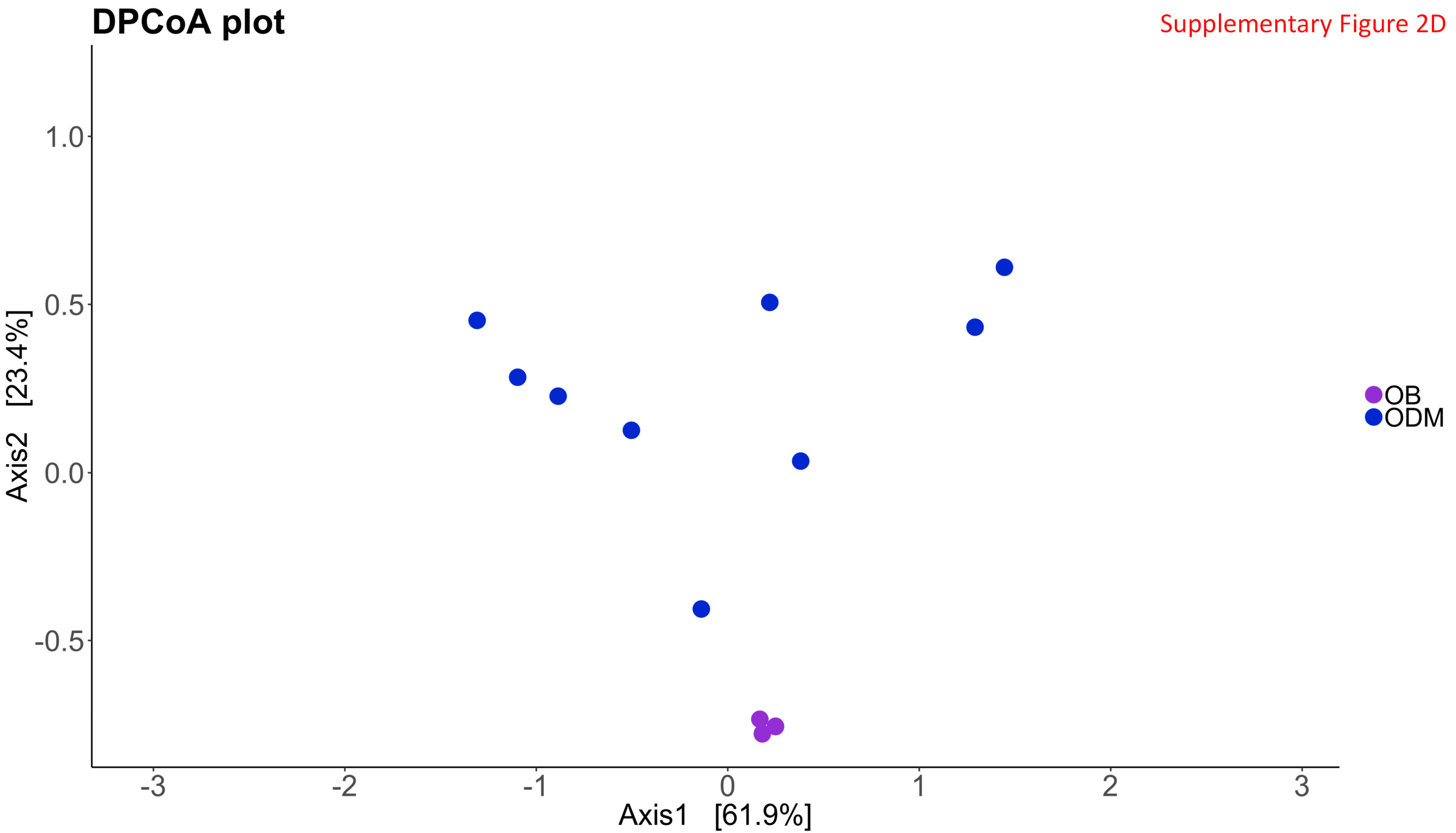
DPCoA plot
Supplementary Figure 2D
1.0
0.5
0.0
-0.5
Axis2 [23.4%]
-3
-2
-1
0
1
2
3
Axis1 [61.9%]
OB
ODM

Supplementary Figure 2E

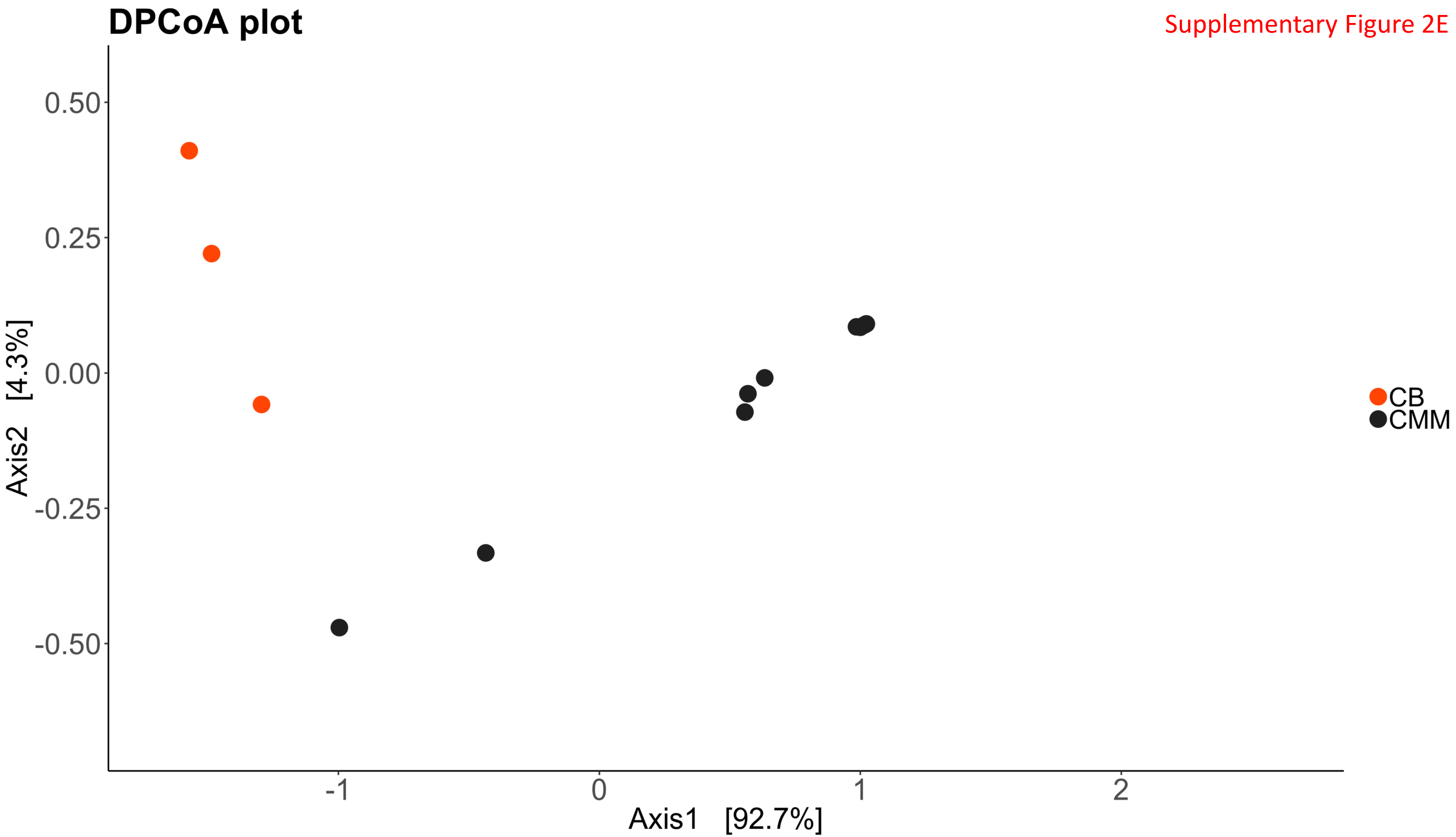

DPCoA plot

Supplementary Figure 2F

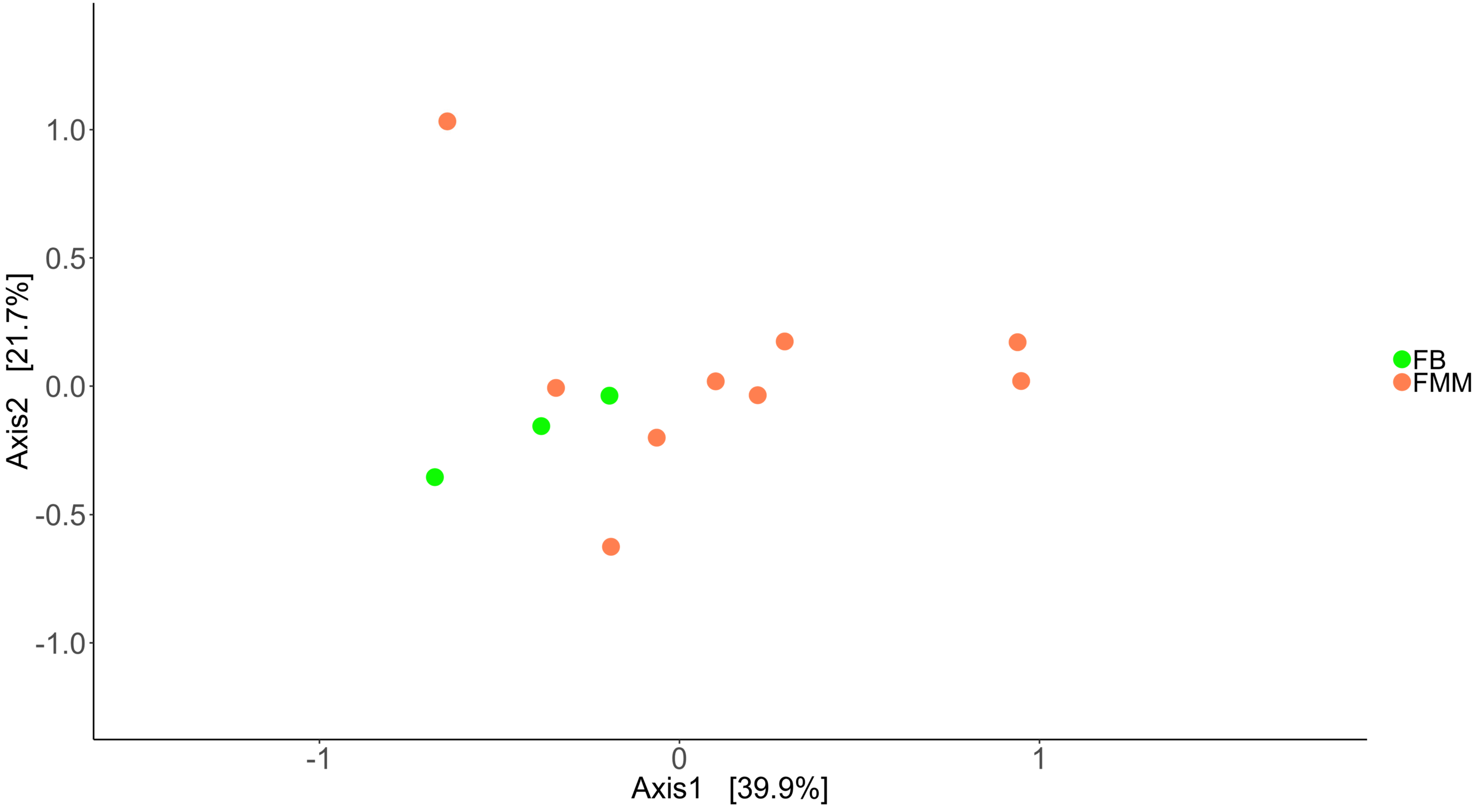

Supplementary Figure 2G

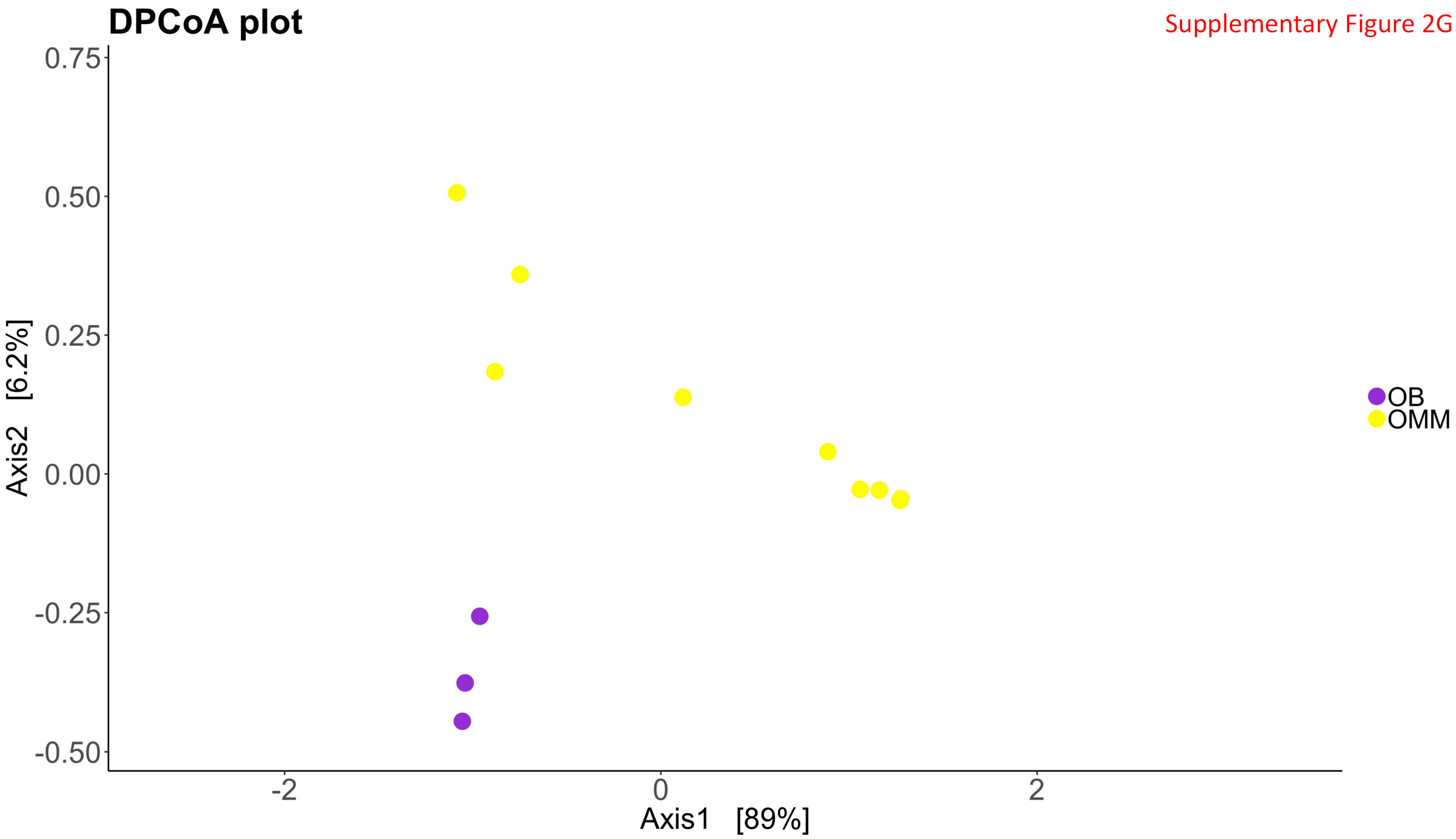

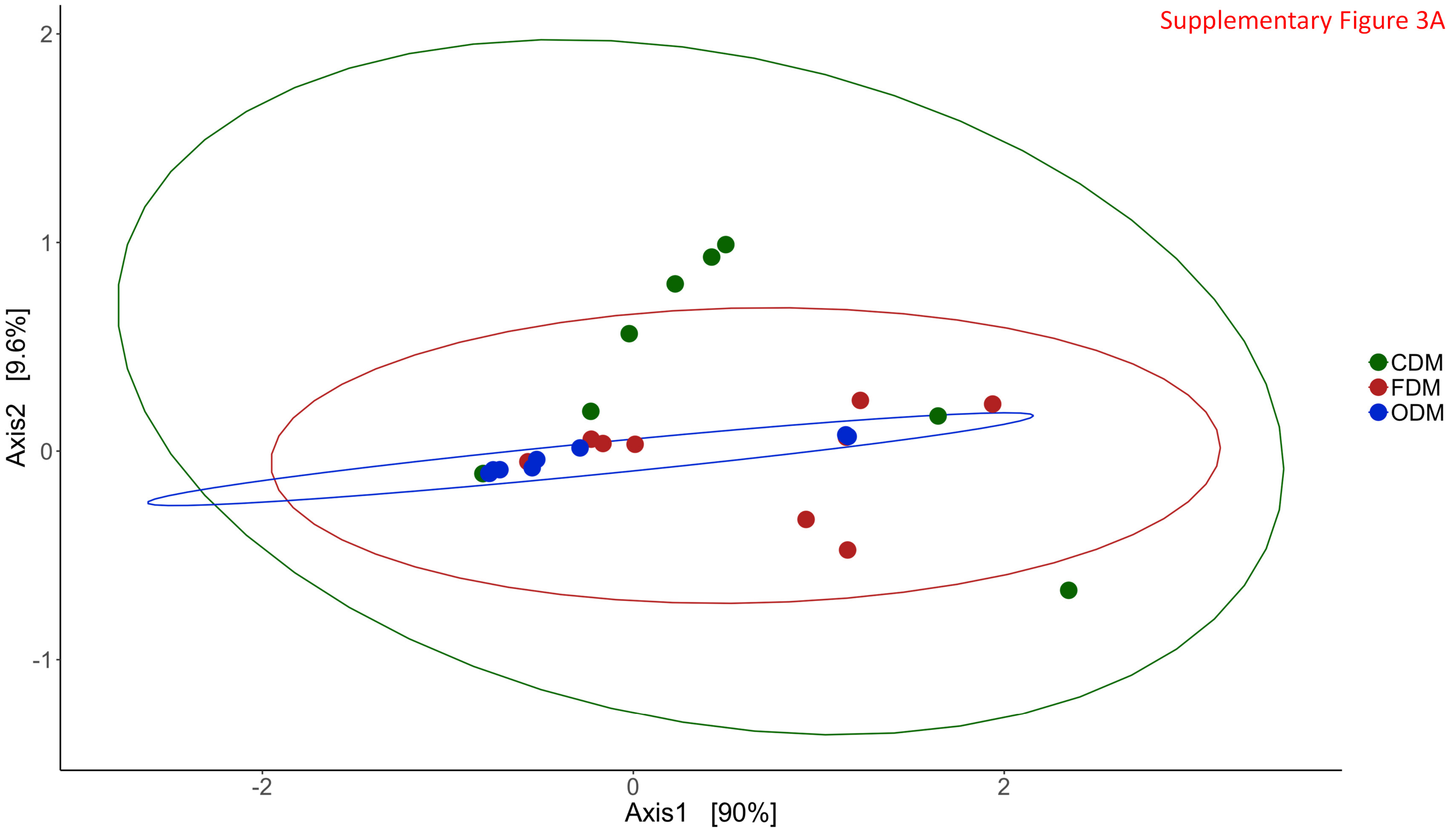

Supplementary Figure 3A
2
1
0
-1
-2
0
2
Axis2 [9.6%]
Axis1 [90%]
CDM
FDM
ODM

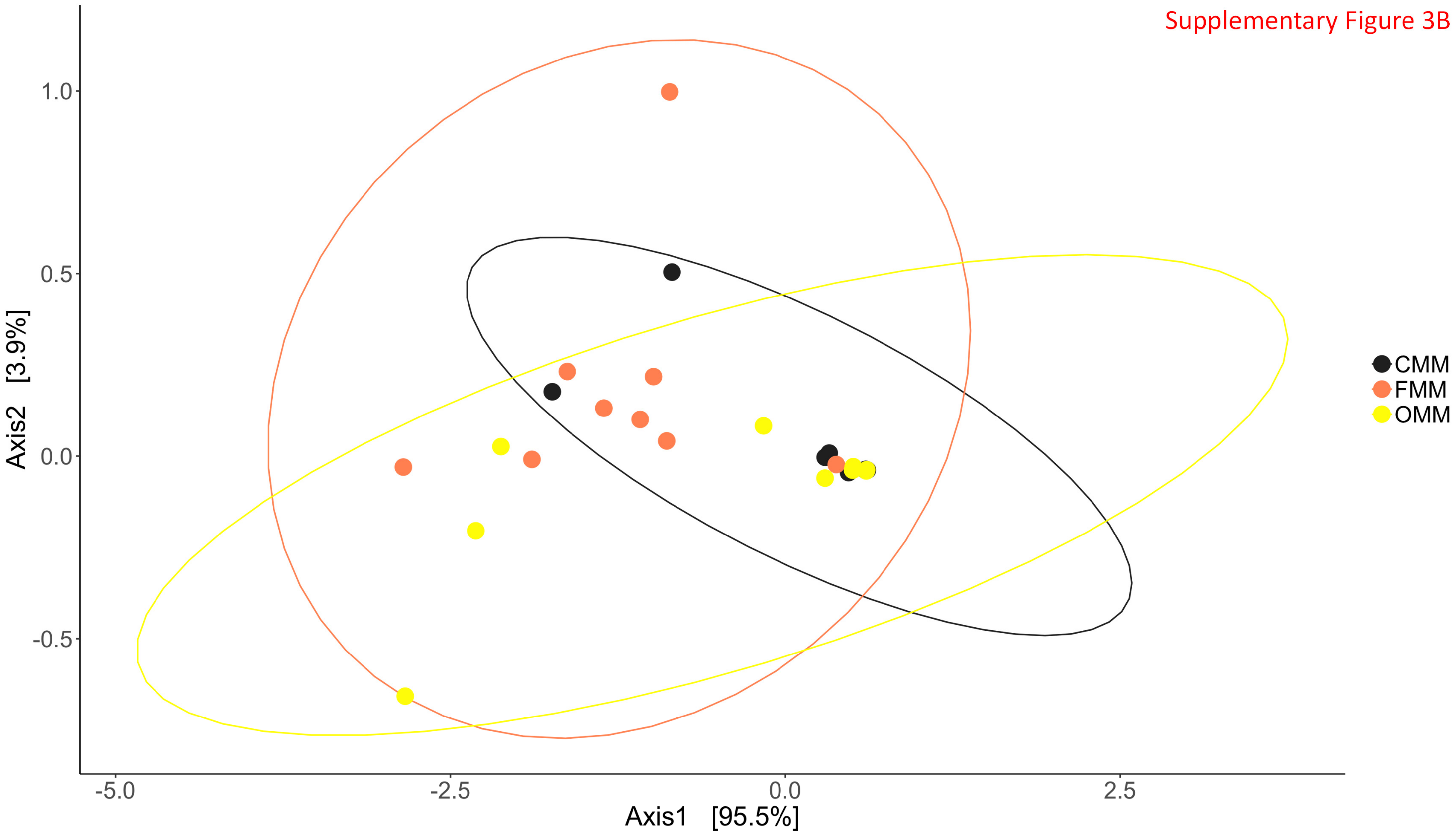
Supplementary Figure 3B
1.0
0.5
0.0
-0.5
Axis2 [3.9%]
-5.0
-2.5
0.0
2.5
Axis1 [95.5%]
CMM
FMM
OMM

Supplementary Figure 4A

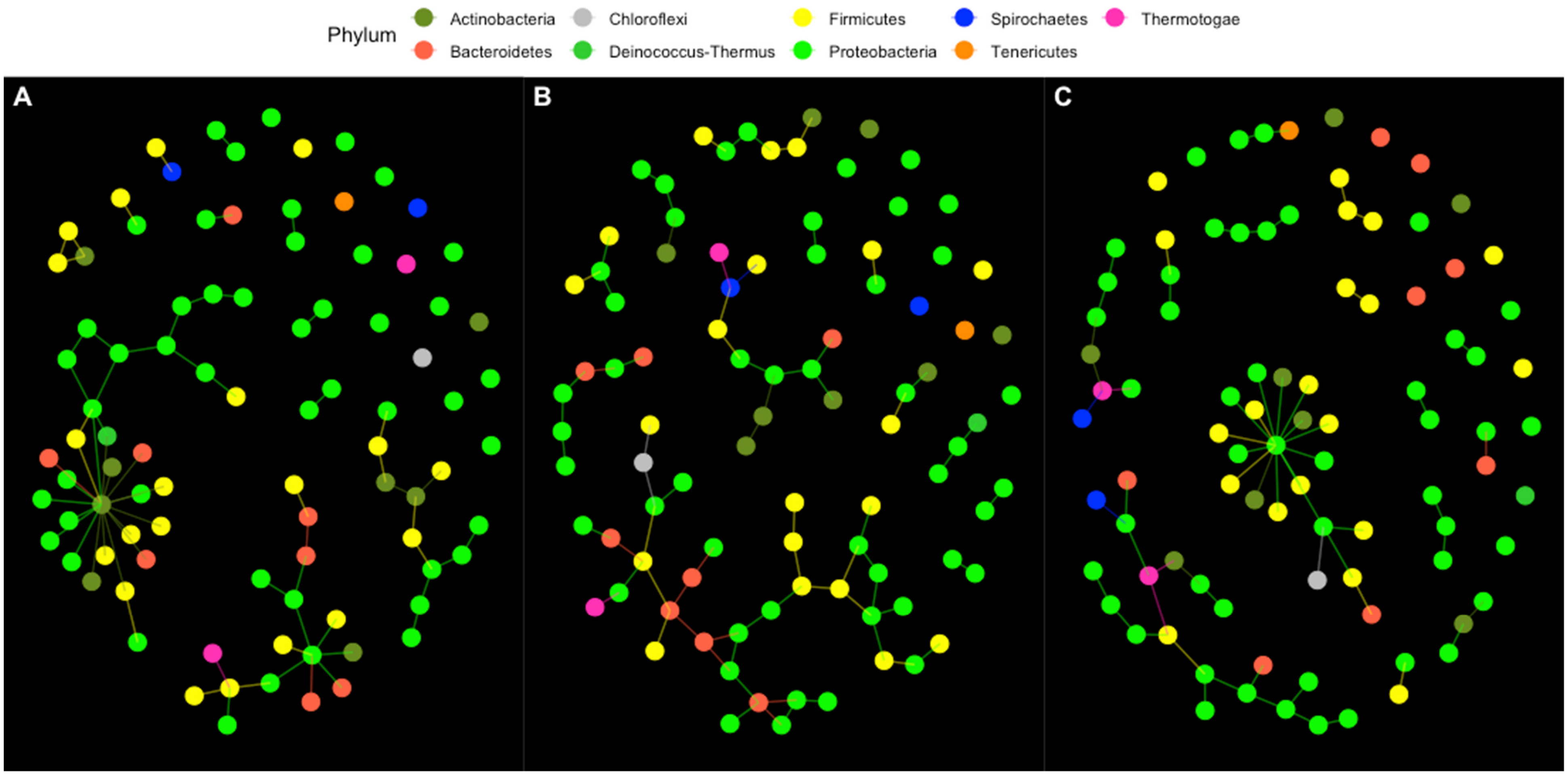

Supplementary Figure 4B

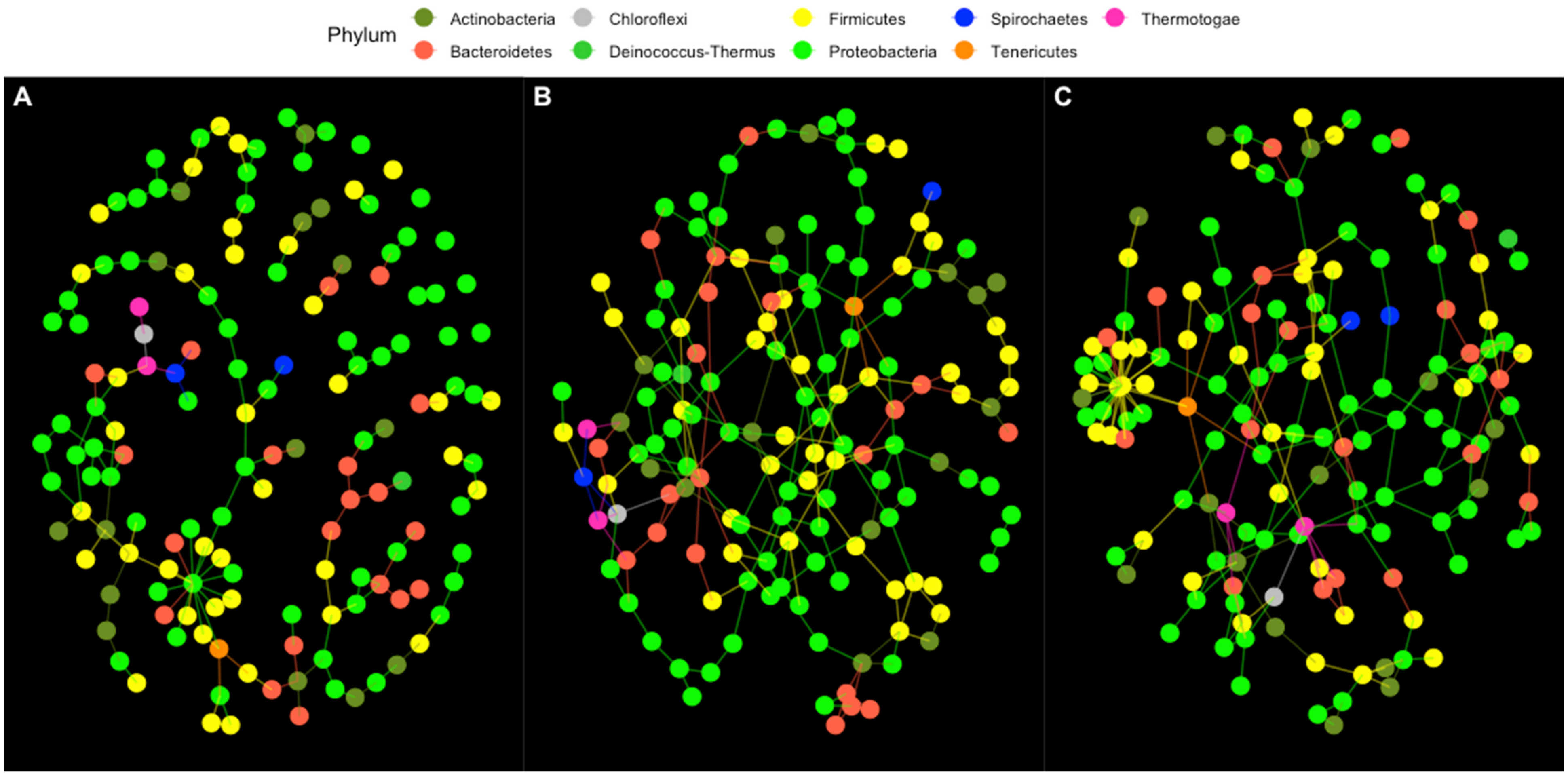

Supplementary Figure 5A

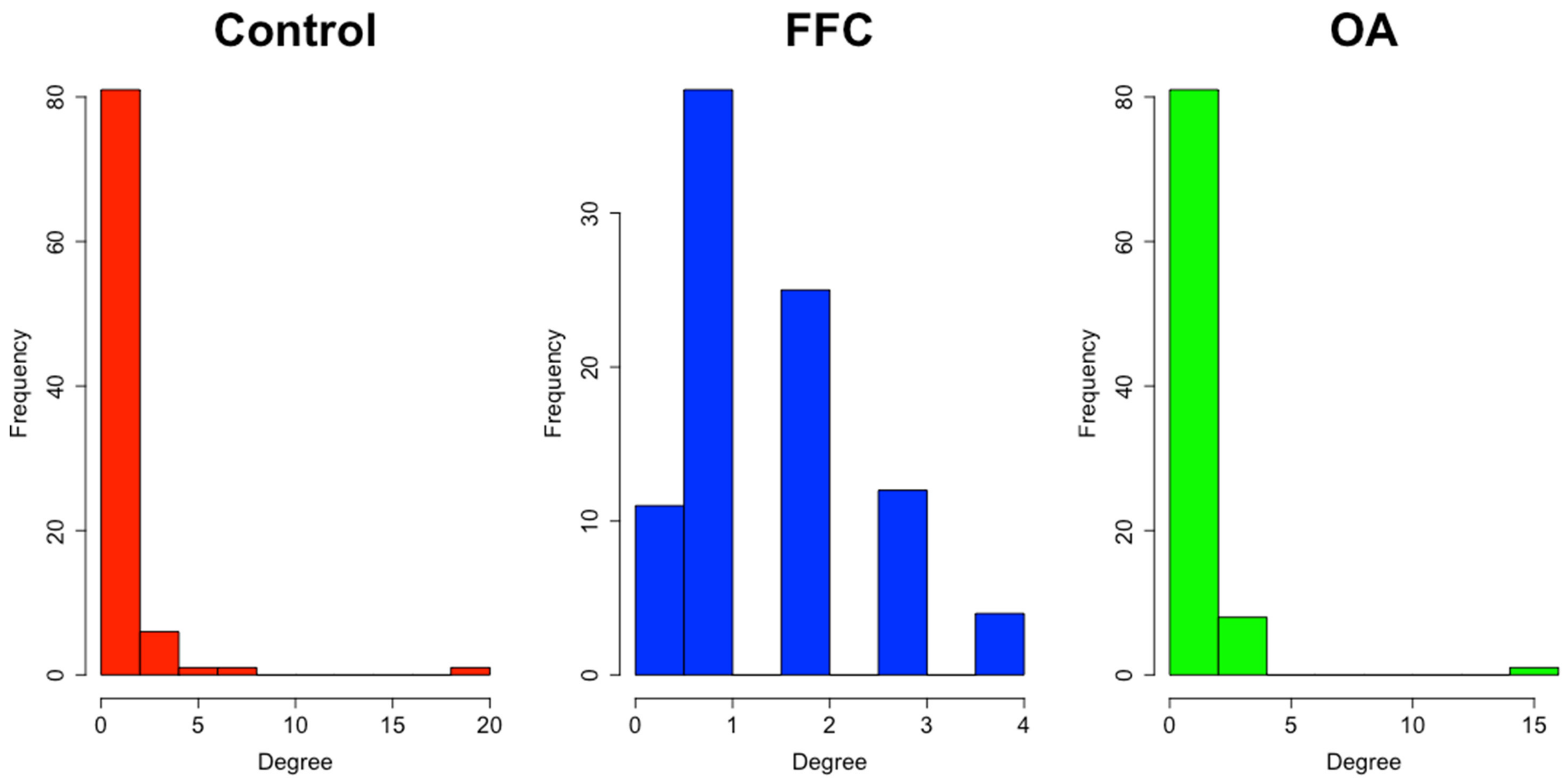

Supplementary Figure 5B

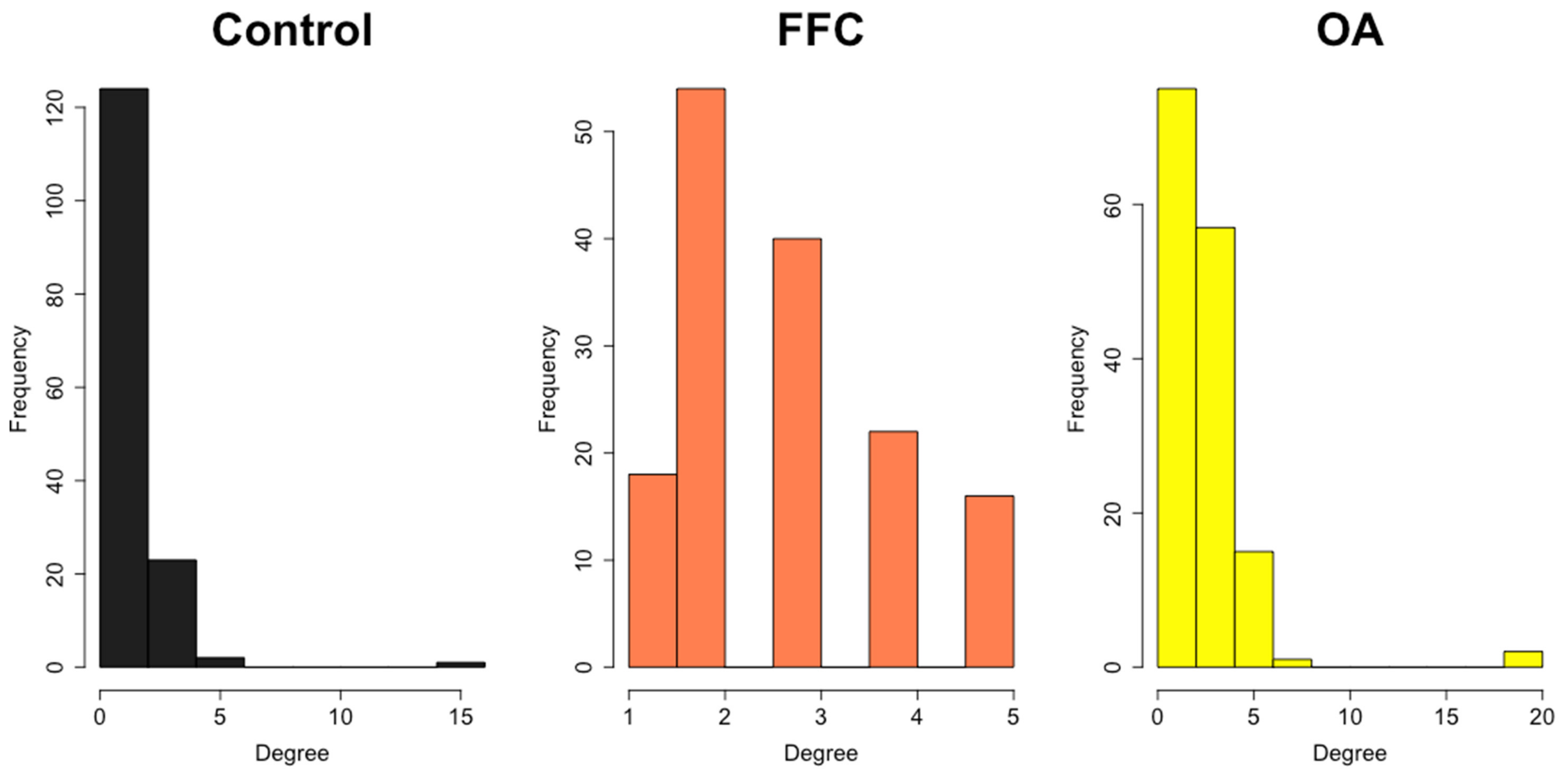

Supplementary Figure 6

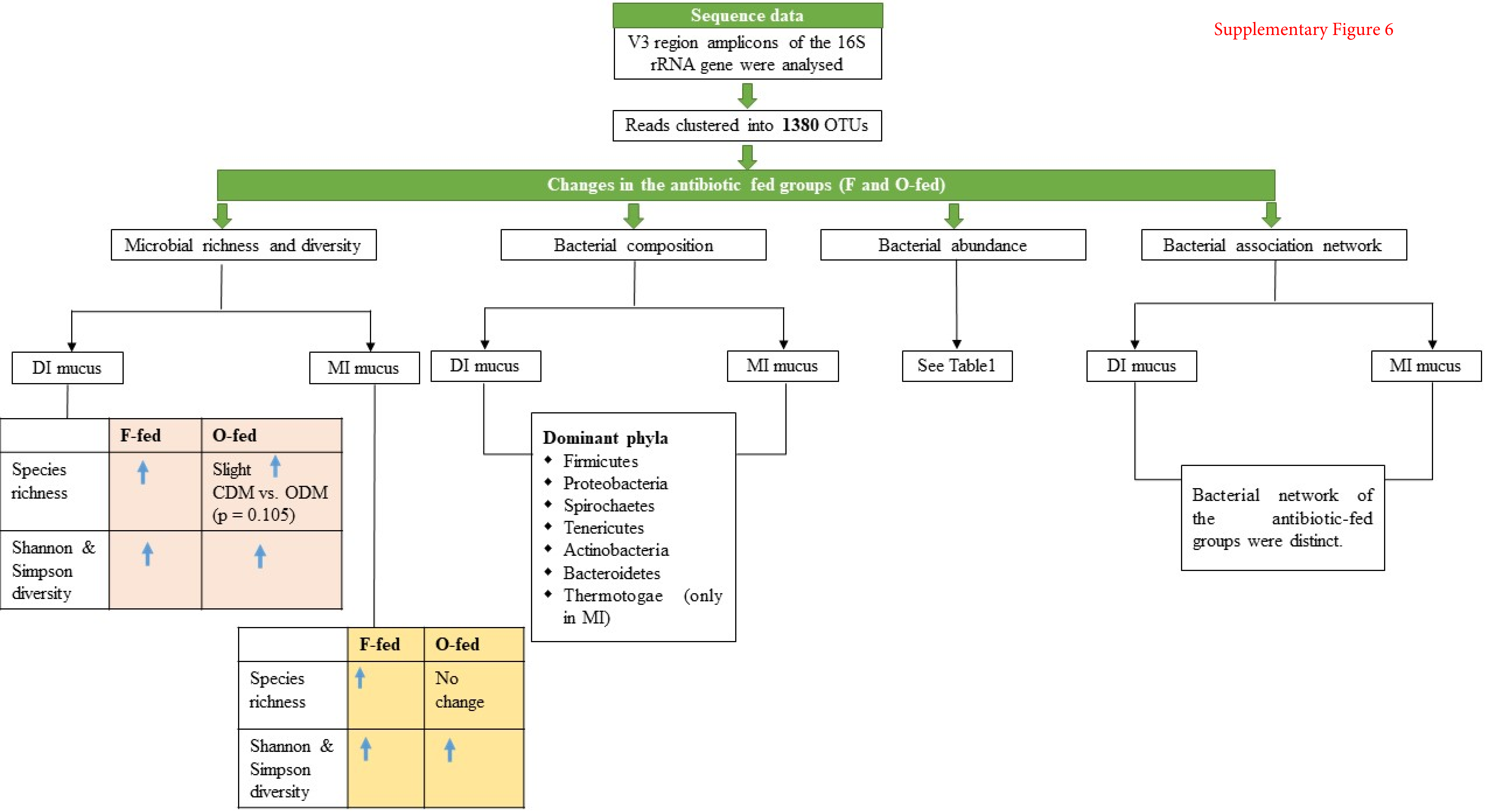

Supplement: Supplementary file 1 [file microorganisms-07-00233-s001.pdf]
